# Insight into the PTEN – p85α interaction and lipid binding properties of the p85α BH domain

**DOI:** 10.18632/oncotarget.26432

**Published:** 2018-12-11

**Authors:** Jeremy D.S. Marshall, Paul Mellor, Xuan Ruan, Dielle E. Whitecross, Stanley A. Moore, Deborah H. Anderson

**Affiliations:** ^1^ Cancer Research Group, University of Saskatchewan, Saskatoon, Saskatchewan, S7N 5E5, Canada; ^2^ Department of Biochemistry, University of Saskatchewan, Saskatoon, Saskatchewan, S7N 5E5, Canada; ^3^ Cancer Research, Saskatchewan Cancer Agency, Saskatoon, Saskatchewan, S7N 5E5, Canada

**Keywords:** PTEN, p85α, regulatory mechanism, lipid binding, structure

## Abstract

The phosphatidylinositol 3-kinase (PI3K) pathway plays a key role in regulating cell growth and cell survival and is frequently deregulated in cancer cells. p85α regulates the p110α lipid kinase, and also stabilizes and stimulates PTEN, the lipid phosphatase that downregulates this pathway. In this report, we determined that the p85α BH domain binds several phosphorylated phosphoinositide lipids, an interaction that could help localize p85α to membranes rich in these lipids. We also identified key residues responsible for mediating PTEN – p85α complex formation. Based on these experimental results, a docking model for the PTEN – p85α BH domain complex was developed that is consistent with the known binding interactions for both PTEN and p85α. This model involves extensive side-chain and peptide backbone contacts between both the PASE and C2 domains of PTEN with the p85α BH domains. The p85α BH domain residues shown to be important for PTEN binding were p85α residues E212, Q221, K225, R228 and H234. We also verified experimentally the importance of PTEN-E91 in mediating the interaction with the p85α BH domain. These results shed new light on the mechanism of PTEN regulation by p85α.

## INTRODUCTION

The phosphatidylinositol 3-kinase (PI3K)/PTEN signaling pathway plays a central role in regulating cell cycle progression, cell growth, migration and survival [1–3]. This pathway is a frequent target of deregulation in many types of cancer through the activation of upstream receptors or via alterations of key PI3K pathway proteins [4]. The p110α catalytic subunit of PI3K phosphorylates phosphatidylinositol 4,5-bisphosphate (PI4,5P_2_) on the 3-position generating phosphatidylinositol 3,4,5-trisphosphate (PI3,4,5P_3_) [5]. This serves as an important signaling lipid which binds proteins such as Akt and PDK1 (3-phosphoinositide-dependent protein kinase 1), recruiting them to the membrane, and facilitating the activation of a cascade of downstream Akt signaling [6]. PTEN is the lipid phosphatase that dephosphorylates PI3,4,5P_3_ back to PI4,5P_2_, switching off Akt signaling. Thus, the balance of PI3K activity and PTEN activity determines Akt signaling output.

The p85α protein serves as a regulatory protein for both p110α and PTEN. Monomeric p85α binds to p110α and stabilizes it, as well as constraining its PI3K activity [5]. In response to upstream receptor tyrosine kinase activation, p85α binds to receptors, relocalizing the p85α - p110α complex to the plasma membrane and relieving the inhibition towards p110α [7, 8]. Disinhibited and membrane localized p110α is then able to phosphorylate its target lipid PI4,5P_2_ to PI3,4,5P_3_ to activate the PI3K pathway.

In response to upstream activation signals, PTEN is dephosphorylated within its regulatory domain, allowing it to form homodimers [9]. The p85α protein can dimerize and these dimers associate with PTEN, protecting it from ubiquitination and proteasomal degradation to stabilize PTEN levels [10–12]. Association of p85α with PTEN also stimulates PTEN lipid phosphatase activity to dephosphorylate PI3,4,5P_3_ back to PI4,5P_2_ to inactivate PI3K pathway signaling [12–14].

Aberrant activation of the PI3K/PTEN pathway can occur in cancer cells via activating mutations in p110α and/or p85α that relieve the inhibitory effects, yet maintain binding to promote p110α stability [8, 15–19]. Additional gain-of-function mutations in p110α can also result in constitutive PI3K activity. Similarly, deletions or loss-of-function mutations of PTEN can compromise the ability of the cell to reduce PI3,4,5P_3_ levels resulting in PI3K pathway activation. Each of these instances results in enhanced downstream Akt signaling and the promotion of cell growth and cell survival [1].

The p85α protein contains 5 domains, an SH3 (Src Homology 3) domain capable of binding proline-rich sequences [20], a BH (break-point cluster region homology) domain, an N-terminal SH2 (Src Homology 2) domain (nSH2), an inter-SH2 domain (iSH2) and a C-terminal SH2 domain (cSH2). It also contains two proline-rich regions, the first is between the SH3 and BH domains (PR1) and the second is between the BH and nSH2 domains (PR2). The two SH2 domains are responsible for binding to phosphorylated tyrosine sites on upstream activators like activated receptor tyrosine kinases [21]. The nSH2 domain and iSH2 coiled-coil region mediate binding of p85α to p110α [15]. In addition, p85α homodimerization is mediated by reciprocal interactions between the SH3 domain of one monomer with the PR1 region on a separate p85α monomer [20], together with interactions between the two cSH2 domains [22] and may also involve BH – BH domain interactions [12, 20, 23]. The p85α BH domain can bind and help downregulate several GTPases like Rab5 with important roles in vesicle trafficking during endocytosis [24–28]. Importantly, the BH domain of p85α, either alone or together with the SH3 domain, also mediates binding to PTEN [13] via a distinct interaction that does not compete with Rab5 binding [12, 29].

In this report, we set out to map the residues in both PTEN and the p85α BH domain that mediate the PTEN – p85α BH domain interaction. This information should help better understand the mechanism by which p85α stimulates PTEN lipid phosphatase activity.

## RESULTS

### Defining key PTEN residues required for p85α binding

**Figure 1:**
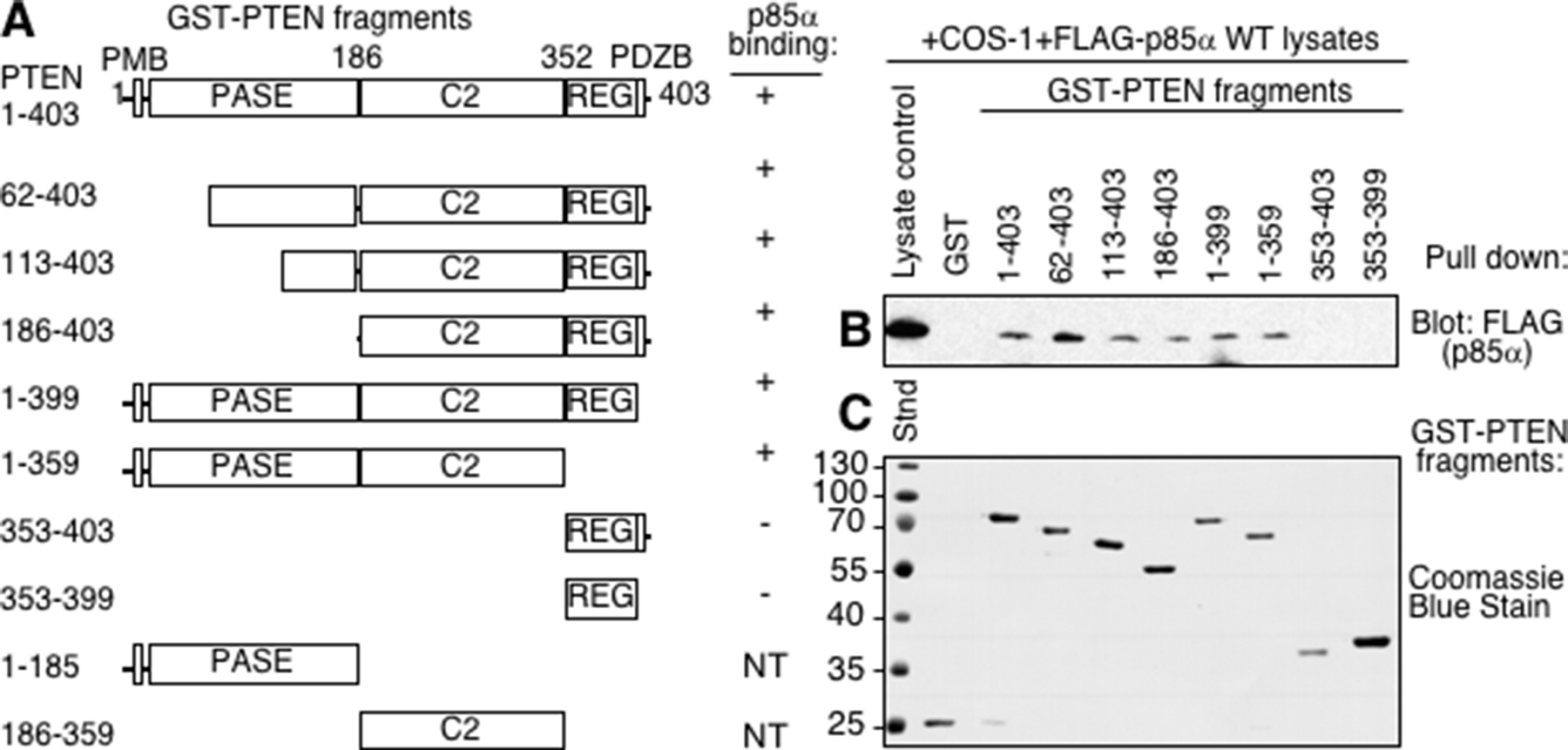
The C2 domain of PTEN is required for p85α binding (**A**) Domains of PTEN expressed as GST-PTEN proteins, immobilized on glutathione Sepharose beads and used in pull-down assay in panel B. PMB = plasma membrane binding domain, PASE = phosphatase domain, C2 = C2 domain, REG = regulatory domain containing key phosphorylation sites, PDZB = PDZ binding domain. Note: the individual PASE and C2 domains were not tested (NT) since the proteins were not stable when expressed. (**B**) The indicated immobilized (GST-)PTEN regions were tested for their ability to bind p85α, supplied from COS-1 lysates expressing FLAG- p85α wild type in a pull-down assay. Bound FLAG (p85α) proteins were detected by immunoblotting with FLAG antibodies. (**C**) Coomassie blue stained gel showing immobilized GST, and GST-PTEN proteins used for panel B.

PTEN consists of multiple domains including the plasma membrane binding region (PMB), the phosphatase domain (PASE), the C2 domain, the regulatory domain (REG) and the PDZ binding region (post synaptic density protein, *Drosophila* disc large tumor suppressor, and zonula occludens-1 protein; PDZB) (Figure 1A). Our previous work using purified proteins demonstrated that PTEN and p85α interact with each other directly [13]. To assess the regions of PTEN required for p85α binding, a series of GST-PTEN fragments were generated. Each PTEN fragment was well expressed in *E. coli* except the individual PASE and C2 domains, such that these latter two were not tested. The majority of the PTEN fragments were able to bind p85α with the exception of the small C-terminal regulatory domain and PDZB regions (Figure 1B–1C). C-terminal deletion of the PDZB and REG domains also did not prevent p85α binding, suggesting that these domains are not required for binding p85α. Progressively larger N-terminal deletions of the PASE domain did not prevent p85α binding, suggesting that it was also dispensable for p85α binding (Figure 1B–1C). All PTEN fragments that bound p85α contained the C2 domain, suggesting that this region of PTEN contained residues important for p85α binding.

**Figure 2:**
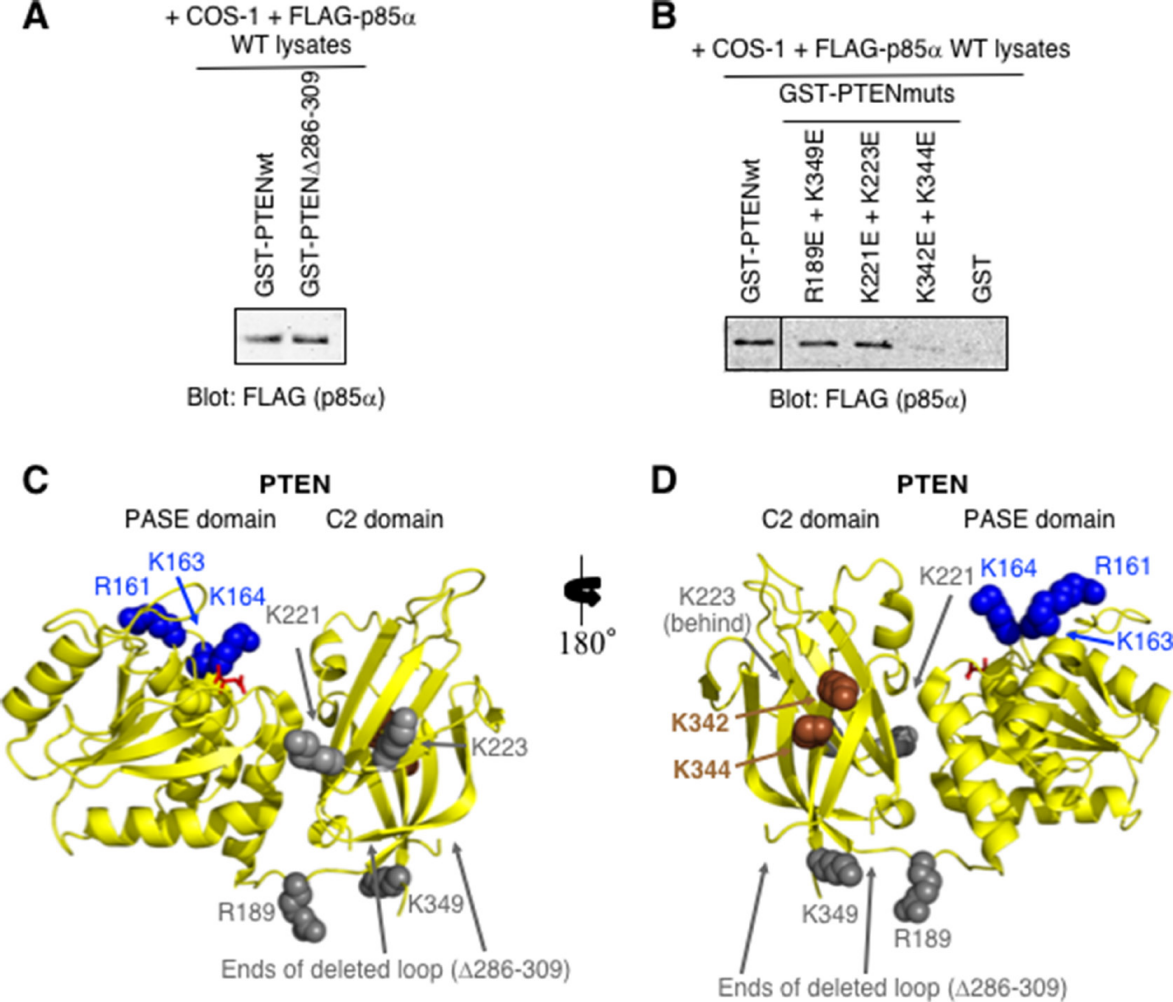
PTEN-K342E+K344E C2 domain mutations within the full-length PTEN are sufficient to prevent p85α binding (**A**) Pull-down immunoblot showing that the deletion of the flexible loop within the context of GST-PTEN (Δ286–309) does not prevent FLAG-p85α binding. (**B**) PTEN C2 domain mutants were tested as immobilized GST-PTEN protein for binding to FLAG- p85α (from COS-1-expressing cell lysates) in a pull-down assay. (**C–D**) Two views of the structure of PTEN-PASE-C2 domains (PDB ID# 1D5R) including amino acid residues 7–353; internal deletion of loop within the C2 domain Δ286–309. The PASE domain basic residues important for membrane association are shown in blue (R161, K163, K164). Mutations within the PTEN C2 domain that reduce (brown; K342, K344), or have no effect (grey) on p85α binding are indicated. L-tartrate is bound in the active site (red).

Analysis of the PTEN C2 domain suggests that it contains a protease-sensitive loop (residues 286–309) as suggested by the fact that it was deleted from the constructs used to obtain the crystal structure [30]. When this loop was deleted from the purified GST-PTEN protein, binding of p85α by the GST-PTENΔ286–309 mutant was similar to that of the wild type PTEN protein, suggesting this region of PTEN is not required for binding (Figure 2A).

Several PTEN C2 domain mutants were then generated and tested for p85α binding (Figure 2B). In each instance a pair of surface exposed basic residues, located close together within the PTEN C2 domain, were both mutated to acidic residues in an attempt to disrupt the binding of p85α (Figure 2C–2D). The PTEN-K342E+K344E C2 domain mutant showed little or no binding of p85α, suggesting that mutation of these residues, within the context of the full-length PTEN protein, is sufficient to block interactions with full-length p85α (Figure 2B). These K342 and K344 residues may also define a surface of PTEN that contacts p85α.

### Defining key p85α BH domain residues required for PTEN binding

**Figure 3:**
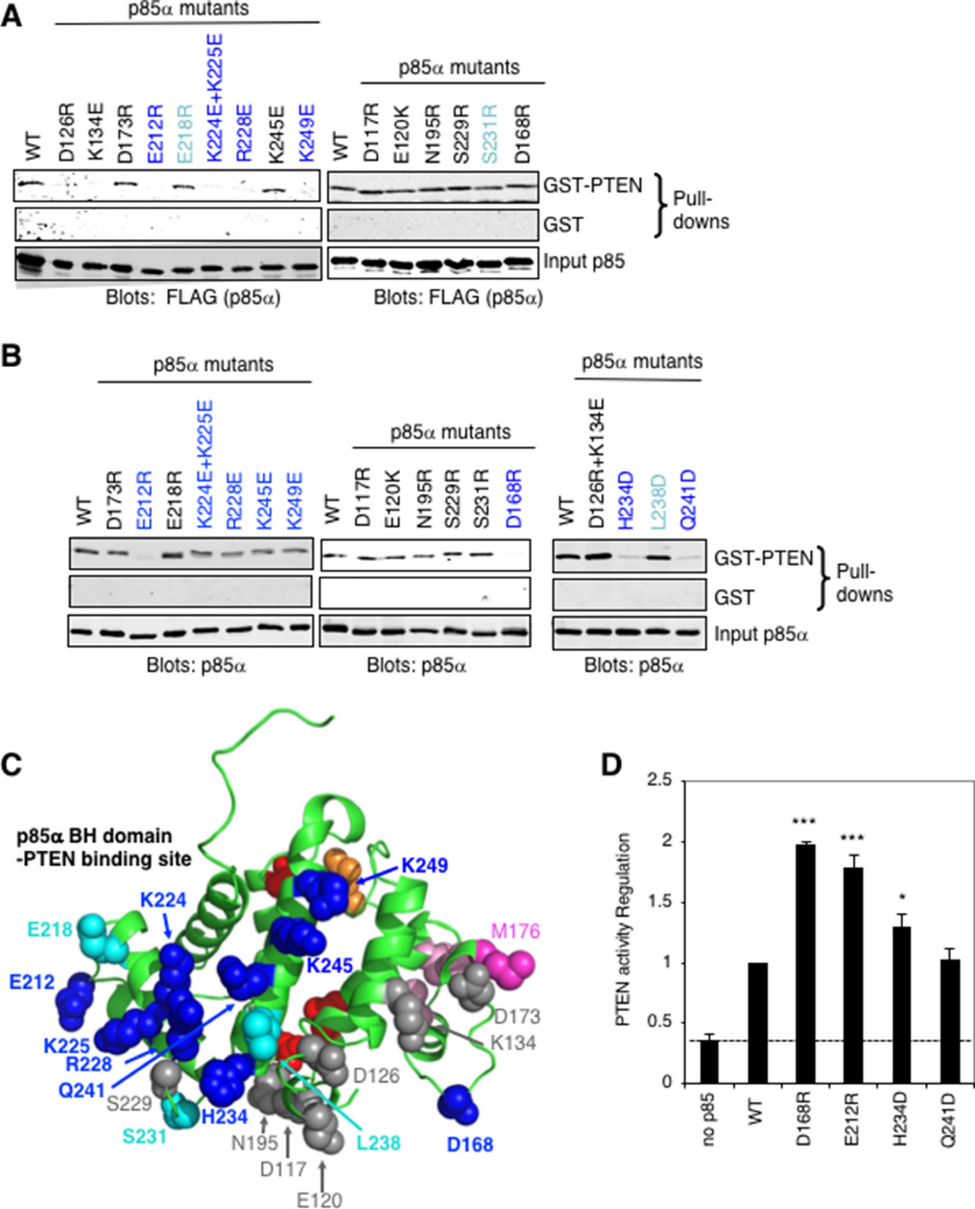
Mapping the PTEN binding site on the p85α BH domain (**A**) GST or wild type GST-PTEN proteins were used in a pull-down assay with the indicated FLAG-p85α mutants from COS-1-expressing cell lysates. (**B**) GST or wild type GST-PTEN proteins were used in a pull-down assay with the indicated purified p85α mutant proteins. Data is representative of 3 to 6 independent binding experiments. (**C**) The p85α BH domain structure (PDB ID# 1PBW) showing the location of p85α mutations with substantial consistent decreased binding to PTEN (dark blue), some decreased PTEN binding (cyan) and those with little or no effect (grey). Red and orange residues mark the Rab5 binding site (around the back; orange: R151 and red: L161, V263, R274), whereas pink residues are important for BH–BH crystal dimerization (light pink: L161, I177, V181 and dark pink: M176). (**D**) PTEN lipid phosphatase activity was measured either alone (no p85α) or with the added p85α wild type or mutant protein. Mean ± SEM from five independent assays. ^***^P < 0.001, ^*^P < 0.05 as compared to wild type p85α. The dashed line indicates PTEN activity in the absence of p85α.

Our initial characterization of the direct binding and regulation of PTEN by p85α showed that the BH domain of p85α was sufficient to mediate binding to PTEN [13]. Using the crystal structure of the human p85α BH domain as a guide (1PBW) [23], we carried out an extensive mutational analysis of the bovine p85α BH domain to define key residues important for mediating binding to PTEN (Figure 3). For this work, two complimentary GST-PTEN pull-down analyses were used. One analysis used lysates from FLAG-tagged p85α mutants expressed in COS-1 cells, which would also contain endogenous p85α protein (Figure 3A). Several p85α mutants showed consistently reduced binding to PTEN, including E212R, K224E+K225E, R228E and K249E. Other p85α mutants showed some reductions in binding to PTEN, though these results were more variable (E218R and S231R). Two p85α mutants (D126R and K134E) showed reductions in PTEN binding, however, in the next analysis, the double mutant D126R+K134E consistently showed robust binding to PTEN (Figure 3B). The proximity of these two residues to each other and the impact of their charge reversals suggested that the two individual mutations created a charge repulsion that was compensated for in the double mutant. Thus, these residues likely do not contribute to direct interactions with PTEN.

A second analysis used purified p85α mutant proteins, expressed in *E. coli* as GST fusion proteins, and cleaved from GST (Figure 3B). For this analysis, several additional mutants were tested including the D126R+K134E double mutant noted above. The p85α mutants that showed marked reductions in binding to PTEN included E212R, D168R, H234D and Q241D, with some reduction in binding for K224E+K225E, R228E, K245E and K249E, and variable results were observed for L238D. The position of the p85α residues tested is illustrated, relative to the residues that form BH–BH domain dimer lattice contacts within the protein crystals, and the recently mapped Rab5/GTPase binding site surface of the BH domain [29] (Figure 3C).

The p85α mutations that had the greatest reduction of steady-state PTEN binding in the second analysis using bacterially expressed and purified p85α protein (D168R, E212R, H234D and Q241D) were selected for further characterization. To assess if the mutations altered the overall folding of the protein, circular dichroism (CD) spectroscopy was used to evaluate possible changes in protein secondary structure (Supplementary Figure 1). The far UV CD spectrum for each of these mutants was similar to that for the wild type p85α protein, suggesting that the point mutations did not severely disrupt protein folding.

PTEN lipid phosphatase activity is positively regulated by p85α binding [12, 13]. To assess the PTEN regulatory activity of the selected p85α mutants, we carried out PTEN lipid phosphatase activity assays using purified PTEN and p85α (Figure 3D). Interestingly, compared to wild type p85α, several of the p85α mutants (D168R, E212R, H234D) showed increased stimulation of PTEN activity. This suggests that reductions in stable interactions between some p85α mutants and PTEN may increase the influence of the p85α mutant. Since these p85α mutants are not sequestered in PTEN complexes as long as the wild type protein, they can interact with more PTEN molecules in a given amount of time, an effect observed previously [29]. Thus, although several of these p85α BH domain mutations decreased binding to PTEN, they did not prevent PTEN activity regulation.

### Identification and characterization of a novel binding site within the p85α BH domain

**Figure 4:**
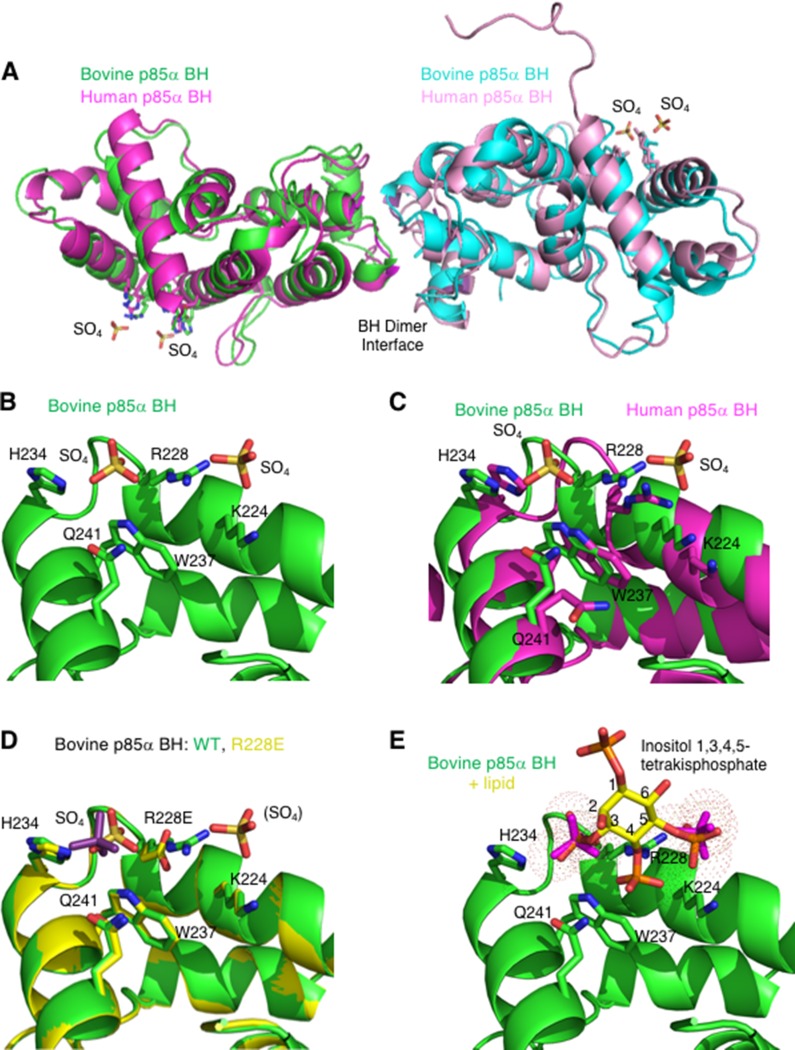
The crystal structure of the bovine p85α BH domain reveals two bound sulfate molecules that may indicate a new binding site (**A**) Overlay of the bovine p85α (105–319) crystal (green and cyan; PDB ID# 6D81; resolution 2.25 Å) with the crystal structure for the human p85α (105–319) protein fragment (magenta and pink; PBD ID# 1PBW; resolution 2.0 Å). The locations of two sulfate molecules per monomer are also indicated. (**B**) View of the potential novel binding site observed in the bovine p85α BH structure. Sulfate molecules and the side chains of amino acids involved in their binding (K224, R228, H234, W237, and Q241) are indicated. (**C**) Overlay of potential binding site for bovine and human p85α. The side chain orientation for residues involved in the potential binding site for both the bovine p85α-BH structure (green), and the previously solved human p85α BH domain (magenta) are shown for comparison. (**D**) Overlay of the potential binding site region of the p85α BH wild type (green) and p85α-BH-R228E engineered mutant (yellow). The one sulfate present in the p85α-BH-R228E structure is coloured purple for differentiation. (**E**) Close up of the potential binding site in p85α-BH with a superimposed inositol 1,3,4,5-tetrakisphosphate, which positions the PO_4_ groups very near the sulfate ions (magenta). Inositol groups are positioned so that phosphates at positions 3 and 5 are located where the electron densities assigned to sulfate ions were present.

We previously reported solving the structure of the bovine p85α BH domain (105–319) [29], which was very similar to the structure of the previously determined human p85α BH domain (105–319) [23] as shown in a structural overlay (Figure 4A). A unique feature we noticed in the bovine p85α BH domain structure that was not described in the previous paper, was clear electron density for two sulfate ions near residues K224, R228, H234, W237, and Q241 (Figure 4B). Sulfate ions were present in the crystallization solution used for the bovine p85α BH domain, whereas the human p85α BH domain crystallization was carried out in a buffer lacking sulfate ions. The residues involved in coordinating the pair of sulfate ions within the bovine p85α BH domain are conserved in the human protein and they adopt similar positions within the structure (Figure 4C), suggesting it may also contain a novel binding site. Further, since the residues involved in coordinating the two sulfate ions were highly conserved between the bovine, human and mouse p85α BH domain sequences, and to some degree in other vertebrates (Supplementary Figure 2), such a novel binding site may also be conserved.

We generated, crystalized and determined the structure of the p85α-BH-R228E protein (2.4 Å), containing a mutation in a potential sulfate binding site residue. In this crystal structure, one of the sulfate ions was clearly absent, and the remaining sulfate ion reoriented itself within the site (Figure 4D and Table 1). No other major structural differences were observed. This result is consistent with R228 contributing to sulfate binding.

**Table 1:** Data collection and refinement statistics for p85α BH domain R228E mutant (PDB ID# 6MRP)

	p85α-BH R228E
**Beamline**	CMCF-ID
**Resolution range (Å)^b^**	46.56–2.40 (2.44–2.40)
**Space group**	P2_1_2_1_2_1_
**Unit cell (a, b, c, α, β, γ)**	85.19, 92.70, 93.12, 90 90 90
**Total reflections**	217767
**Unique reflections^b^**	29393 (1454)
**Multiplicity^b^**	7.4 (7.5)
**Completeness^b^ (%)**	100.0 (100.0)
**R-merge^a,b^**	0.085 (0.601)
**Mean I/sigma (I)**	33.0 (4.84)
**Refinement**	
**Resolution range (Å)^b^**	46.56–2.40 (2.489–2.403)
**Reflections ^b^**	29340 (2850)
**R-work^b,c^**	0.1841 (0.2146)
**R-free^b,d^**	0.2247 (0.2481)
**Number of non-hydrogen atoms**	2955
**macromolecules**	2848
**Number solvent atoms**	107
**Protein residues**	360
**RMS bonds (Å)**	0.012
**RMS angles (°)**	1.235
**Ramachandran favoured (%)**	96.51
**Ramachandran outliers (%)**	0.87
**Rotamer outliers (%)**	0.62
**Wilson B-factor**	40.39

^a^$R_{\text{merge}}=\sum_{\mathrm{hkl}}\sum_{i}|<I{\left(\mathrm{hkl}\right)}_{\mathrm{obs}}>-I{\left(\mathrm{hkl}\right)}_{\mathrm{obs},i}|/\sum_{\mathrm{hkl},i}I{\left(\mathrm{hkl}\right)}_{\mathrm{obs},i}\;\text{where}\;I{\left(\mathrm{hkl}\right)}_{\mathrm{obs},i}$ is the individual measurement of an hkl intensity and $<I{\left(\mathrm{hkl}\right)}_{\mathrm{obs}}>=\sum_{i}I{\left(\mathrm{hkl}\right)}_{\mathrm{obs},i}/N$ ; where i = 1 to N individual reflections are measured.

^b^Values in parentheses correspond to the highest resolution shell.

^c^$R_{\text{work}}=\sum_{\mathrm{hkl}}\left|\left|F_{\mathrm{obs}}\left(\mathrm{hkl}\right)\right|\right|-\left|F_{\mathrm{calc}}\left(\mathrm{hkl}\right)\right|\left|/\sum_{\mathrm{hkl}}|F_{\mathrm{obs}}\left(\mathrm{hkl}\right)\right|,\text{where}\left|F_{\mathrm{obs}}\left(\mathrm{hkl}\right)\right|\text{and}\left|F_{\mathrm{calc}}\left(\mathrm{hkl}\right)\right|$ are the observed and calculated amplitudes, respectively, for the structure factor F(hkl).

^d^R_free_ is the equivalent of R_work_ for 5% of the reflections (randomly selected) which were not used in structure refinement.

The p85α protein is known to be a key regulatory protein of the PI3K/PTEN pathway; one of its mechanisms acting through binding to PTEN and facilitating the PTEN-mediated dephosphorylation of PI3,4,5P_3_ to PI4,5P_2_ [13]. We recognize that sulfate binding to Arg is very common in proteins crystallized from ammonium or lithium sulfate solutions, but the distance between the two sulfate positions (7.1 Å) is similar to that of the two PO_4_ groups (6.8 Å) in inositol 1,3,4,5-tetrakisphosphate, raising the intriguing possibility that this surface may recognize phospholipid head groups. This is modeled in Figure 4E and suggests that the p85α BH domain could bind phosphoinositide head groups.

### The p85α BH domain binds to phosphorylated phosphoinositol lipids

**Figure 5:**
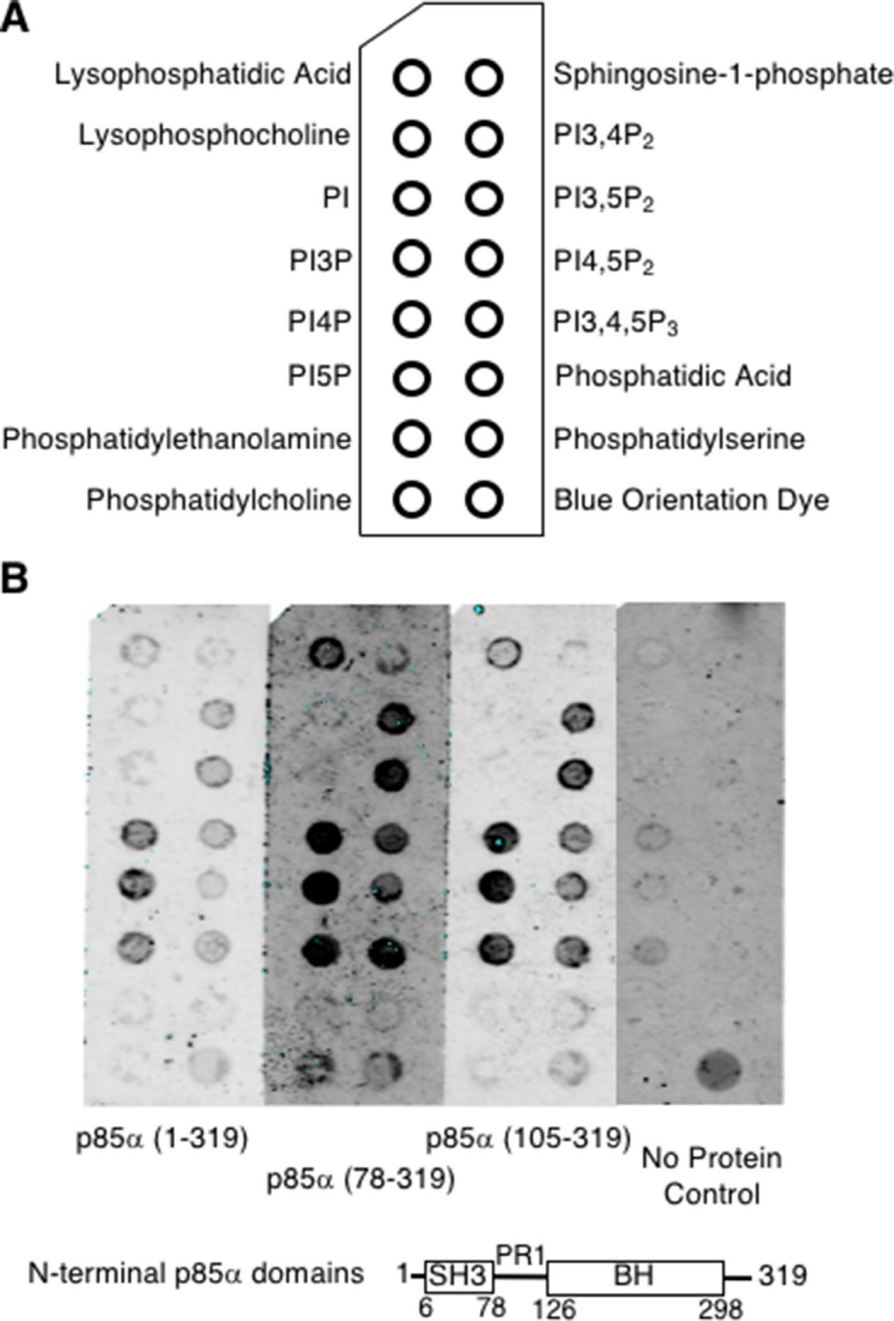
The p85α-BH domain binds directly to lipids, particularly phosphorylated PIs (**A**) PIP strip nitrocellulose membrane with spotted lipids (100 pmol per spot). (**B**) PIP strips were blocked and probed with the indicated purified p85α protein (37 nM). Bound proteins were detected with an anti-p85α BH antibody, followed with an infrared secondary antibody and visualized using a LICOR Odyssey infrared scanner. PI = phosphatidylinositol; PI3P = phosphatidylinositol 3-phosphate; PI4P = phosphatidylinositol 4-phosphate; PI5P = phosphatidylinositol 5-phosphate; PI3,4P_2_ = phosphatidylinositol 3,4-bisphosphate; PI3,5P_2_ = phosphatidylinositol 3,5-bisphosphate; PI4,5P_2_ = phosphatidylinositol 4,5-bisphosphate; PI3,4,5P_3_ = phosphatidylinositol 3,4,5-trisphosphate.

To assess the lipid binding ability of the p85α BH domain, several p85α BH domain-containing protein fragments, p85α (1–319), p85α (78–319), or p85α (105–319), were used to probe lipid blots (Figure 5A). Bound p85α protein was detected using p85α BH domain-specific antibodies (Figure 5B) [13]. All three p85α fragments showed binding to the following lipids: (PI3P, PI4P, PI5P) > (PI3,4P_2_, PI3,5P_2_, phosphatidic acid) > (PI4,5P_2_, PI3,4,5P_3_) > lysophosphatidic acid. Binding of p85α BH protein to the lipids immobilized on the blots was also found to be concentration dependent (Supplementary Figure 3). The p85α (1–319) protein fragment showed less signal but the same binding profile. Since all three p85α fragments were able to bind to these lipids, this demonstrates that the BH domain in isolation is capable of lipid binding. All lipids bound by the p85α BH domains were negatively charged and contained phosphate groups, whereas we observed little binding to non-phosphorylated lipids such as PI, uncharged lipids or those without phosphates. These results raise the interesting possibility that the potential new binding site seen within the p85α BH domain can bind lipids containing phosphate groups.

**Figure 6:**
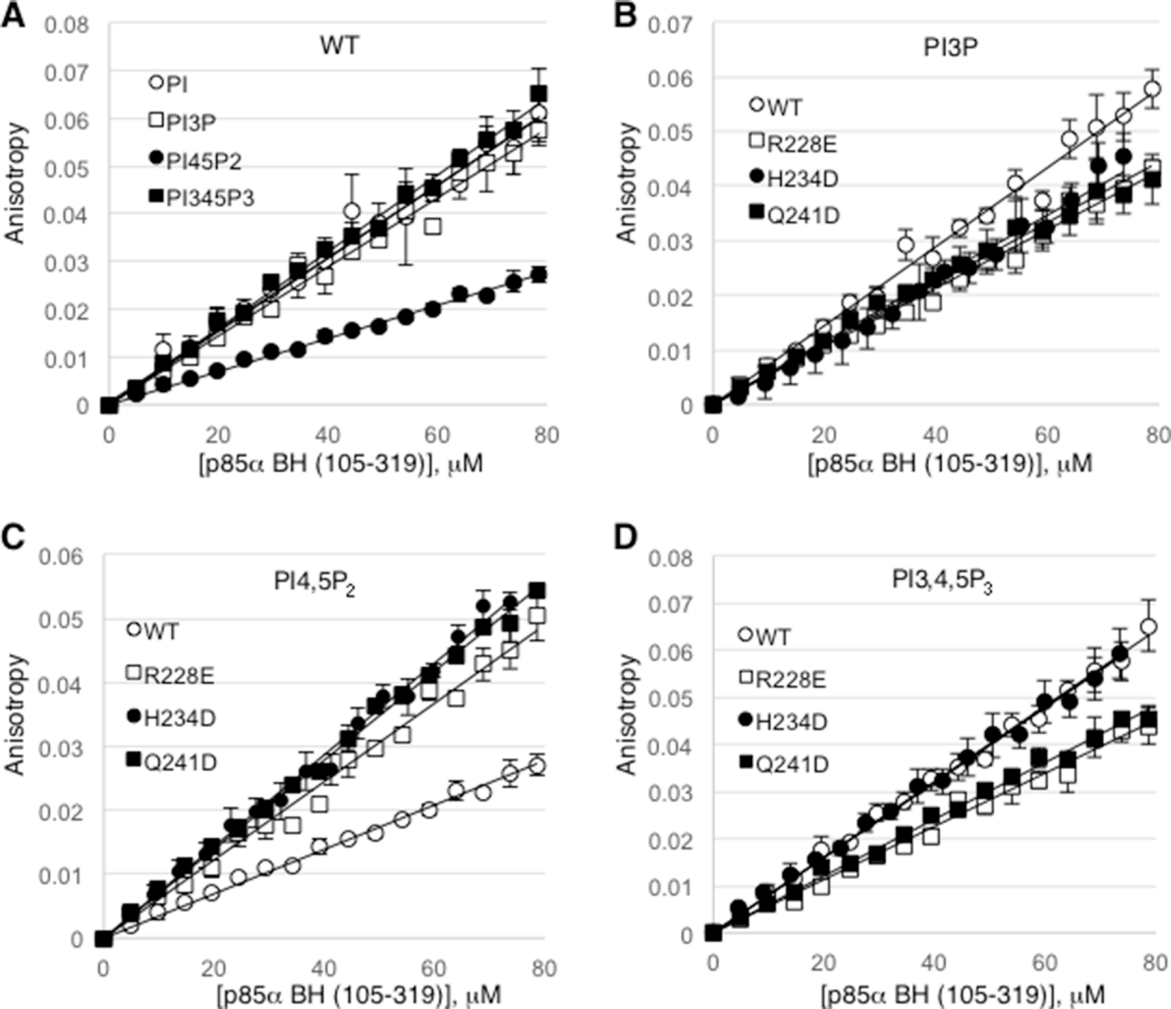
Rotational anisotropy analysis of the lipid binding affinities for the wild type p85α BH domain and several p85α BH domain mutants (**A**) Changes in fluorescence polarization were measured for Bodipy-FL labelled phosphatidylinositol lipids: phosphatidylinositol (PI), phosphatidylinositol 3-phosphate (PI3P), phosphatidylinositol 4,5-bisphosphate (PI4,5P_2_), and phosphatidylinositol 3,4,5-trisphosphate (PI3,4,5P_3_), upon mixing with increasing concentrations of wild type p85α BH (105–319) protein. (**B–D**) Changes in fluorescence polarization for the indicated bodipy-labeled lipid were measured for the wild type or p85α BH (105–319) mutant proteins as in panel A; PI3P (B), PI4,5P_2_ (C), PI3,4,5P_3_ (D). Mean ± SEM is shown for each data point from 3 independent experiments.

To measure the binding affinity of the p85α BH domain (105–319) for specific phospholipids, we carried out rotational anisotropy experiments using fluorescently labeled short-chain lipids (Figure 6). In general, the p85α BH domain bound very weakly to these lipids and we did not reach saturation even at the highest protein concentrations tested of 79 µM. Thus, the binding may be extremely weak and/or the interaction may have a very fast off rate. Based on the slopes of these binding interactions, the wild type p85α BH domain bound equally well to PI, PI3P and PI3,4,5P_3_, whereas the binding to PI4,5P_2_ was about 2-fold weaker (Figure 6A). We also tested 3 p85α BH domain mutant proteins, each with a single point mutation in a residue within the potential binding site (R228E, H234D and Q241D). All 3 p85α mutants bound PI3P with similar affinities, which were reduced as compared to the wild type protein (Figure 6B). In contrast, all 3 mutants bound to PI4,5P_2_ better than the wild type protein (Figure 6C). For PI3,4,5P_3_, the H234D mutant showed a similar binding affinity as the wild type protein, whereas the R228E and Q241D mutants had a reduced affinity (Figure 6D). These results suggest that although the p85α BH domain binds to lipids, it does so with a very low affinity. Moreover, we have not established strong specificity, indicating that the lipid binding properties of the p85α BH domain is worthy of further investigation.

### Modeling the PTEN – p85α BH domain complex

Our extensive mutational analysis identified a number of key p85α BH domain residues important for binding of PTEN, including: D168, E212, E218, K224, K225, R228, S231, H234, L238, Q241 and K249 (Figure 3A–3B). Similarly, PTEN residues K342 and K344 were found to be important for binding p85α (Figure 2B). In addition, recent data suggested that although p85α dimerization is required for PTEN binding, the BH domain of p85α may not dimerize in solution [22]. Instead LoPiccolo *et al.* suggest that p85α dimerization is most likely mediated by SH3 domain – proline-rich region interactions [20, 22], as well as a recently described second intermolecular interaction mediated by the cSH2 domains [22].

Taking all of this information into consideration, docking studies were performed using the ClusPro 2.0 server [31–34], inputting the human PTEN crystal structure (PDB ID# 1D5R) and a monomer (A chain) from our recently solved bovine p85α BH domain (105–319) crystal structure (PDB ID# 6D81) [29]. The residues of the p85α BH domain and PTEN experimentally determined to be important to mediate the PTEN – p85α BH interaction as noted above were set as attraction residues. Several promising models were obtained that all included many of the experimentally determined p85α BH domain residues found to be important in mediating binding to PTEN. To select the most probable model, we reasoned that the PTEN – p85α BH domain complex should be highly conserved for both the human and bovine p85α proteins so we independently repeated the docking analysis using the human PTEN crystal structure and a monomer (A chain) from the human p85α BH domain (105–319) crystal structure (PDB ID# 1PBW).

**Figure 7:**
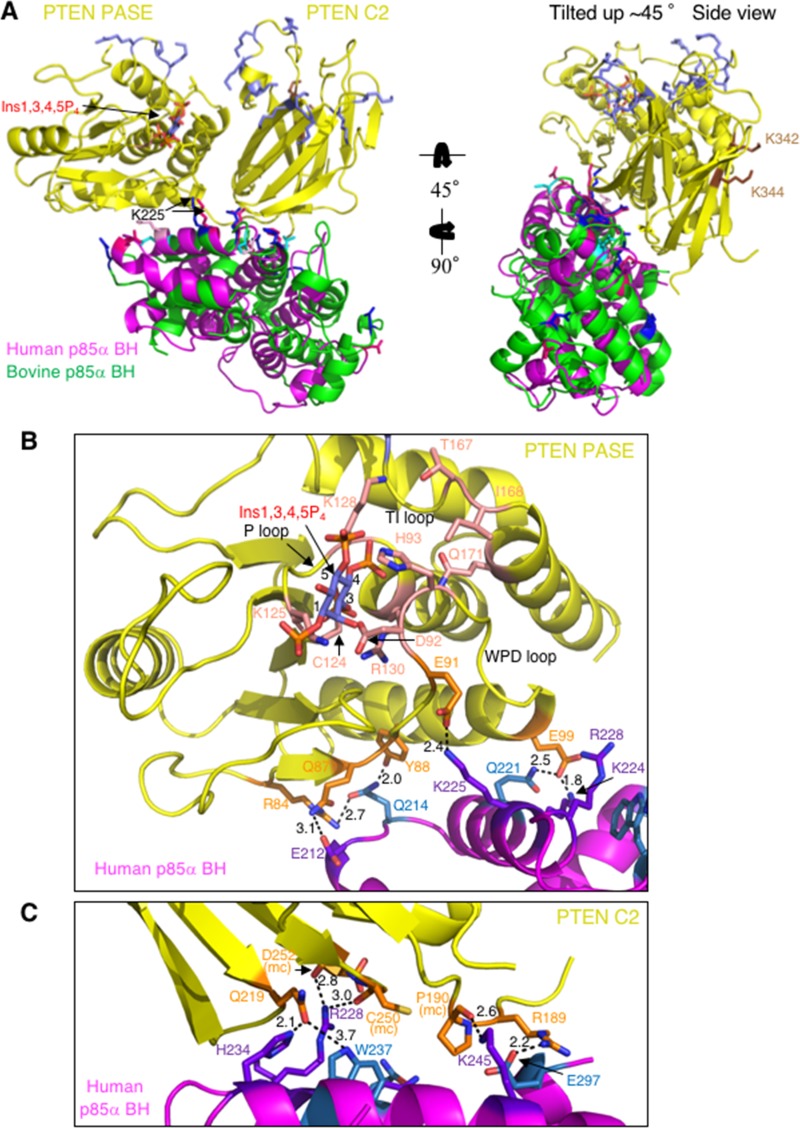
Docking model for human or bovine p85α BH domain monomers and PTEN, incorporating the new experimental results regarding important residues in each protein for mediating their binding (**A**) Both the C2 and PASE domains of PTEN (yellow; PDB ID# 1D5R) make side-chain and main chain contacts with the human p85α BH domain (magenta; PDB ID# 1PBW) and bovine p85α BH domain (green; PDB ID# 6D81). PTEN active site is modeled with bound inositol 1,3,4,5-tetrakisphosphate (Ins1,3,4,5P_4_; blue-orange-red) and the active site C124 is shown in salmon. Basic residues of PTEN important for membrane binding (slate blue across the top) and p85α binding (brown; K342 and K344; located in the C2 domain around the back) are also shown. The bovine p85α BH domain (green) residues shown experimentally to be important for PTEN are shown blue (dark blue – consistent binding results; cyan – variable binding results). The human p85α BH domain (magenta) residues corresponding to those important for PTEN are shown (dark pink – consistent binding; light pink – variable binding). (**B**) Close-up of the contacts between the human p85α BH domain (magenta) and the PTEN PASE domain (yellow; PTEN C2 domain is hidden). PTEN residues involved in making direct contacts with the human p85α BH domain are shown in orange, with the active site residues shown in salmon with a bound Ins1,3,4,5P_4_ lipid head group. The human p85α BH domain residues involved in direct side-chain contacts with PTEN that were also experimentally tested and determined to be important for PTEN binding are shown in purple, with additional contact residues identified by the docking model shown in sky-blue. Bond distances are shown in Å. (**C**) Close-up of the contacts between the human p85α BH domain (magenta) and the PTEN C2 domain (yellow; PTEN PASE domain is hidden). Residues are colored as in panel B. mc = main chain.

One model emerged from both docking analyses and is the most consistent with available experimental binding data for PTEN and p85α (Figure 7A). This model involves extensive side-chain and peptide backbone contacts between both the PASE and C2 domains of PTEN and the p85α BH domains (Figure 7B–7C, Supplementary Figure 4, and Supplementary Table 1) with a buried surface area of 1211 Å^2^ (PTEN – bovine p85α BH) and 1366 Å^2^ (PTEN – human p85α BH). The p85α BH domain residues that directly contact PTEN in the two docking models were not identical, however both models implicated E212, Q221, K225, R228, H234 and W237. Of these residues, four were part of the input data for the docking analysis, previously shown to be important for PTEN binding (E212, K225, R228 and H234) (Figure 3), whereas Q221 and W237 are new residues not previously tested.

In support of this model, many of the p85α BH domain residues involved in direct side-chain contacts with PTEN in the docking model are highly conserved across the p85α sequences from a variety of vertebrates (Supplementary Figure 2). This model for the PTEN – p85α BH complex suggests that both the PASE domain of PTEN (Figure 7B; Supplementary Figure 4A) and the C2 domain of PTEN (Figure 7C, Supplementary Figure 4B and Supplementary Table 1) make a similar number of contacts with the p85α BH domain. The PTEN residues involved in direct side-chain contacts with the p85α BH domain in the docking models are highly conserved across the PTEN sequences from a variety of vertebrates (Supplementary Figure 5), consistent with them serving an important role in the binding interface of PTEN and p85α. The two PTEN residues found experimentally to be important for p85α binding are not located within the interface of the PTEN – p85α BH complex, raising the possibility that they form contacts with other regions of p85α, since full-length p85α proteins were used in the PTEN mutational analysis. Several of the p85α BH domain residues that can bind a pair of sulfate ions are also involved in mediating interactions with PTEN (Figure 7C; Supplementary Figure 4B), suggesting that if phosphorylated lipids bind to the p85α BH domain at this location, binding of lipid and PTEN would be mutually exclusive.

### Role of key p85α BH domain residues near the PTEN active site

**Figure 8:**
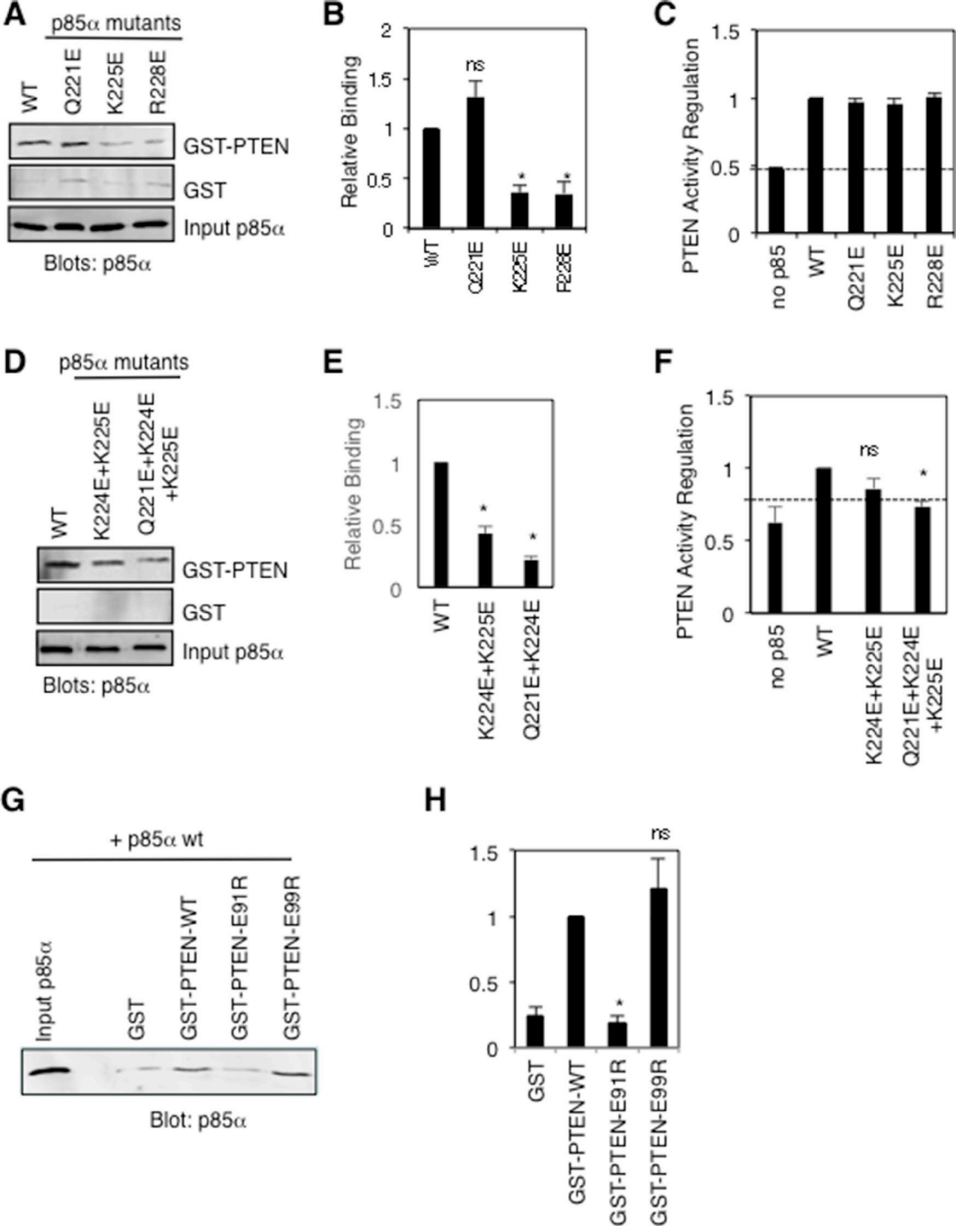
Mutational analysis of key p85α and PTEN residues proposed to make important contacts between these proteins (**A**) Pull-down binding analysis using GST-PTEN or GST and purified p85α wild type or mutant proteins as in Figure 3B. Similar results were obtained in five independent experiments. (**B**) Quantification of GST-PTEN pull-down results from panel A; mean ± SEM from 5 experiments. ns = not significant; ^*^P < 0.001 as compared to wild type p85α. (**C**) PTEN lipid phosphatase activity was measured either alone (no p85α) or with the added p85α wild type or mutant protein. Mean ± SEM from five independent assays. The dashed line indicates PTEN activity in the absence of p85α. (**D**) Pull-down binding assays as in panel A for double and triple p85α mutants. Similar results were obtained in five independent experiments. (**E**) Quantification of GST-PTEN pull-down results from panel D; mean ± SEM from 5 experiments. ^*^P < 0.001 as compared to wild type p85α. (**F**) PTEN activity assay as in panel C. Mean ± SEM from five independent assays. ns = not significant; ^*^P < 0.001 as compared to wild type p85α. The dashed line indicates PTEN activity in the absence of p85α. (**G**) Pull-down binding analysis using GST (control) and GST-PTEN wild type or mutant proteins and purified p85α as in Figure 3B. Similar results were obtained in five independent experiments. (**H**) Quantification of GST-PTEN pull-down results from panel G; mean ± SEM from 5 experiments. ns = not significant; ^*^P < 0.001 as compared to wild type PTEN.

Since p85α has been shown to stimulate PTEN lipid phosphatase activity [12, 13], we took a closer look at the active site of PTEN to assess possible contributions from the docked p85α BH domain. Interestingly, in both docking models p85α-K225 is predicted to make important side-chain interactions with PTEN-E91, and p85α-Q221 is predicted to interact with PTEN-E99. PTEN residues E91 and E99 are located on the ends of the WPD loop containing D92 and H93, residues that directly contribute to the lipid phosphatase activity of PTEN [30]. Therefore, we generated individual p85α mutations Q221E (new) and K225E, to test mutation of K225 as a single mutation since the initial analysis tested K224E+K225E within the same protein. We also included the R228E mutant in this analysis, as a p85α BH domain mutation that reduced PTEN binding, with unknown effects on PTEN lipid phosphatase activity regulation. The docking model predicts that p85α-R228 makes contacts with peptide backbone main-chains C250 and D252 within the PTEN C2 domain. Both the p85α-K225E and p85α-R228E mutants showed significant reductions in PTEN binding without altering their ability to stimulate PTEN lipid phosphatase activity, whereas the p85α-Q221E mutant behaved like the wild type p85α protein (Figure 8A–8C). Since the Q221E p85α mutation does not appear to disrupt PTEN binding, we speculate that p85α-K224 can still interact productively with PTEN-E99, as seen in the model (Figure 7B). We went on to test the PTEN binding and PTEN lipid phosphatase activity regulation of both the p85α-K224E+K225E double mutant and p85α-Q221E+K224E+K225E triple mutant (Figure 8D–8F). Both the double and triple p85α mutants showed significant reductions in PTEN binding, with the triple mutant also showing a corresponding reduction in its ability to stimulate PTEN activity. These results suggest that p85α residues Q221, K224 and K225 contribute to the binding and regulation of PTEN activity.

We also generated several new PTEN mutations within the modeled interaction interface, predicted to be important for p85α binding (R84E, E91R, E99R, Q219R). The R84E and Q221R mutations rendered these GST-PTEN proteins unstable, preventing us from assessing their p85α binding ability. The PTEN-E99R protein retained p85α binding ability, whereas the PTEN mutation E91R caused a significant reduction in p85α binding, providing additional support for our model (Figure 8G–8H).

## DISCUSSION

Our PTEN deletion analysis suggested that the C2 domain of PTEN was involved in mediating binding to p85α. A previous report showed that the PTEN PASE domain may also be sufficient to mediate binding to p85α [12]. Since this latter analysis was carried out using coimmunoprecipitations in a more complex mammalian cell system, that also expresses endogenous PTEN, and PTEN can homodimerize, these results are more challenging to interpret [12]. Our model for PTEN – p85α BH domain complex formation implicates both the C2 and PASE domains in mediating contacts with the p85α BH domain, suggesting that both regions of PTEN may be involved in p85α binding.

Pairs of point mutations within the PTEN C2 domain demonstrated that mutation of K342E+K344E caused a marked reduction in the binding of full-length p85α. The model for PTEN – p85α BH domain docking does not position K342 and K344 at the interface of this contact surface. Thus, we speculate that these mutations may exert their effects either indirectly through allosteric changes in PTEN structure and/or via disrupting interactions between PTEN and other domains of p85α. PTEN mutations K342N (endometrial cancer) and K344R (prostate cancer) [35] have been reported in the Catalogue of Somatic Mutations in Cancer (COSMIC) database. This suggests the possibility that these mutations may disrupt p85α binding and regulation of PTEN activity that may contribute to enhanced PI3K pathway activation as seen in many cancers.

Our extensive mutagenesis of the p85α BH domain and subsequent PTEN binding analysis identified a number of residues that are important for PTEN binding. One analysis used mammalian cell lysates containing p85α mutants, whereas a second analysis used p85α mutant proteins purified from *E. coli*. The results obtained were similar though not identical, possibly due to the more complex environment within the cell lysates that allows for post-translation modifications and/or associations with cellular proteins, including dimerization with wild type p85α. The p85α BH domain mutations that showed reduced PTEN binding included D168R, E212R, K224E+K225E, R228E, H234D and Q241D. Some of these p85α mutations were also tested for PTEN activity modulation and, unexpectedly, several showed increased stimulation of PTEN lipid phosphatase activity (D168R, E212R and H234D). The reason for this result is not clear, but we speculate that these p85α mutants with reduced contacts between p85α and PTEN, may alter the relative orientation of the two proteins to impact PTEN lipid phosphatase reaction rates. It is also possible that the addition of p85α may help stabilize PTEN or could help solubilize the lipid substrate and thus, contribute to the increase in PTEN activity. The PTEN binding site is located on the opposite face of the p85α BH domain as compared to the GTPase/Rab5 binding site, consistent with previous reports that these sites are distinct [12, 29].

We also identified a new lipid binding function for the p85α BH domain, with a preference for phosphorylated PI lipids such as PI3P and PI3,4,5P_3_. We used short-chain fluorescently labeled lipid head groups in an attempt to measure the affinity of lipid binding to the p85α BH domain. The affinity of these small lipids was extremely low, indicating that the short chain lipids do not interact with the p85α BH domain in solution, as they do when long chain lipids are used immobilized on a lipid blot, or possibly as part of a lipid bilayer. It is also possible that the interaction requires two adjacent lipids, as would be found on the surface of a membrane, to interact with two p85α BH domains within a p85α-p85α homodimer to produce sufficient avidity.

The region within the p85α BH domain that includes residues K224, R228, H234, W237 and Q241 binds two sulfate groups that are spaced similarly to two of the PO_4_ groups within inositol 1,3,4,5-tetrakisphosphate (Figure 4E), suggesting lipid binding could be accommodated at this site. Our results however do not convincingly address the location of the lipid binding within the p85α BH domain. If this region is involved in lipid binding and the PTEN – p85α BH docking model is correct, lipid and PTEN would compete for p85α BH domain binding, since these sites overlap. A recent report showed that PTEN specifically binds PI3P through the CRB3 loop within the C2 domain of PTEN and that PTEN is associated with microtubules, tethered to PI3P-containing vesicles [36]. This led to the suggestion that the termination of downstream Akt signaling on endosomes may be linked in part to the recruitment of PTEN to PI3P-containing endosomes. It is possible that the weak binding of the p85α BH domains to PI3P lipids, identified in this study, may also help localize p85α to vesicles containing high concentrations of PTEN, where p85α can subsequently bind and stimulate PTEN activity.

The large number of p85α BH domain mutations that reduced PTEN binding provided a significant amount of experimental evidence for the location of the PTEN binding site on the surface of the p85α BH domain. Although p85α dimerization is required for PTEN binding [12], recent evidence suggests that although p85α BH domains can form dimers in the crystal lattice [30], BH domains of p85α may not dimerize in solution [22]. Instead, p85α dimerization may be mediated by SH3 domain – proline-rich region interactions [20] and also by a recently described intermolecular cSH2 domain interaction [22]. Thus, a model for the PTEN – p85α BH domain complex was generated using this experimental evidence in the context of a p85α BH domain monomer.

The model indicates that both the C2 and PASE domains of PTEN make extensive contacts with the p85α BH domain, consistent with our data in this report (C2 domain contributions; Figure 1) and a previous report (PASE domain contributions) [12]. A significant number of side-chain and main-chain contacts mediate the interaction, generating a large buried surface area. The p85α BH domain and PTEN contact residues are highly conserved across vertebrates, suggesting an evolutionarily conserved function.

We further evaluated this model by generating and testing additional p85α and PTEN mutants. Several p85α mutations significantly reduced PTEN binding by ∼75%, including K225E, R228E and K224E+K225E, with little or no effect on PTEN activity regulation. Significantly, the p85α-Q221E+K224E+K225E triple mutant showed both a marked reduction in PTEN binding and PTEN lipid phosphatase activity regulation. Mutation of PTEN residue E91 (to R) also significantly reduced p85α binding. These results support the idea that these p85α and PTEN residues may help position the WPD loop of PTEN, containing catalytically important residues K92 and H93, and provide insight into the mechanism by which p85α binds and regulates PTEN.

Our docking model also provides sufficient space for other PTEN and p85α domains not present in the modeled fragments, as well as interactions with other important binding partners. The N- and C-termini of both PTEN and p85α BH domain can be accommodated (PTEN: N-terminus PMB domain, C-terminus REG and PDZB domains; p85α: N-terminus SH3 domain, C-terminus nSH2+iSH2+cSH2 domains) (Supplementary Figure 6). In addition, the model allows the p85α BH domain to simultaneously bind PTEN and GTPases like Rab5 [12, 27, 29], and for PTEN to associate with the plasma membrane via the clusters of basic residues along one surface [30, 36–38] (Supplementary Figure 6). The PTEN – p85α docked model also allows for homodimerization of p85α BH domains [20, 30] and of PTEN PASE domains [9, 39] (Supplementary Figure 6).

A previous report carried out modeling using a similar docking strategy [12], but without the benefit of the p85α BH domain mutations and PTEN binding analysis. The resulting docked model for PTEN – p85α BH domain implicated I127, I133 and E137 in mediating PTEN binding. Subsequently, a triple mutant p85α-I127A+I133A+E137A showed a modest (35%) decrease in PTEN binding [12], suggesting that their contributions to PTEN binding may be fairly small. The position of these residues within our PTEN – p85α BH domain model raises the possibility that they may associate with the PTEN C-terminal regulatory region (not present in the model), which could extend along that surface of the p85α BH domain, perhaps as far as D168 (that our results suggest may be important for PTEN binding), near the BH – BH dimerization site (Supplementary Figure 6B). Therefore, our new model is consistent with the currently available data for both PTEN and p85α, including that in previous reports.

Maintaining sufficient p85α to form p85α − PTEN complexes may have important implications for cancer development and progression. The incidence of intestinal polyps is increased two-fold in PTEN(^+/-^)/p85α(^+/-^) mice compared to PTEN(^+/-^) mice [40]. Mice with a liver-specific deletion of *Pik3r1*(^-/-^), the gene encoding p85α, not only showed decreased PTEN activity, elevated PI3,4,5P_3_ levels and increased Akt activation, they also developed liver tumors which progressed to metastatic cancer [41]. Ablation of p85α was sufficient to drive cancer progression in this model. Under-expression of p85α was also shown to be an indicator of poor prognosis in breast cancer [42]. Thus, reduction of p85α levels, a positive regulator of PTEN activity and function, sufficiently below normal could promote cancer susceptibility and progression, particularly in cells with reduced PTEN. Similarly, mutations within p85α that prevent PTEN interaction and the positive regulation of PTEN activity would also effectively reduce the PTEN tumor suppressor function. With the identification of the PTEN binding site on the p85α BH domain and a model for the PTEN – p85α BH domain complex, mutations of either protein that disrupt complex formation may be oncogenic. In support of this suggestion, the COSMIC database lists mutations in several p85α BH domain residues that are important for PTEN binding that have been identified in human tumors, including D168Y, Q221E and W237L, raising the possibility that they may contribute to tumor formation. PTEN residues implicated in contacting the p85α BH domain have also been found to contain mutations in the COSMIC database, including D84G, Q87P, Y88C (also H/N/S), E91Q (also G/A), E99K and R189K.

In summary, we have identified key residues within the p85α BH domain that define the PTEN binding site. We have used this experimental data to model the PTEN – p85α BH domain complex, and further tested this model by p85α mutagenesis and subsequent measurement of PTEN binding and lipid phosphatase activity regulation. The results of this study suggest that the p85α BH domain makes extensive contacts with PTEN and that critical p85α residues Q221, K224 and K225 play key catalytic roles in the stimulation of PTEN activity. The model sheds new light on the mechanism of PTEN regulation by p85α.

## MATERIALS AND METHODS

### Plasmids and mutagenesis

Generation of the GST-PTEN plasmid has been described [13]. The PTEN deletion mutants were generated by PCR amplification of the human GST-PTEN cDNA and subcloned into pGEX6P1 (Amersham Biosciences) as described previously [43]. GST-PTEN fragments had the following sizes: 1–403 (74 kDa), 62–403 (66 kDa), 113–403 (60 kDa), 186–403 (52 kDa), 1–399 (73 kDa), 1–359 (69 kDa), 353–403 (32 kDa), 353–399 (32 kDa), 1–185 (48 kDa), 186–359 (47 kDa) and Δ286–309 (71 kDa). The GST-p85α and FLAG-p85α and plasmids have been described [27, 44] and contain full-length bovine p85α (residues 1–724), fused in-frame after GST or a triple FLAG epitope tag, respectively. Bovine p85α has 96.8% sequence identity with the human p85α protein. The GST-p85α BH domain (105–319) encoding plasmid has been described [29] and upon expression and PreScission cleavage from GST yields a 24 kDa protein. All GST-p85α proteins were cleaved from GST prior to use in experiments. Site-directed mutagenesis of PTEN and p85α were carried out using the QuikChange method (Agilent), according to the manufacturer’s directions. DNA sequencing to ensure that no unwanted mutations had been introduced verified the entire PTEN and p85 coding regions for each clone.

### Pull-down experiments and immunoblots

Pull-down assays with immobilized GST-PTEN were allowed to bind cell lysates containing FLAG-p85α or purified p85α protein (after cleavage from GST) in blocking as previously detailed [29]. For experiments using FLAG-p85α, COS-1 cells (American Type Culture Collection, CRL-1658) were transiently transfected with FLAG-p85α using lipofectamine (Invitrogen) according to the manufacturer’s directions. Cells were lysed in 1 ml lysis buffer per 10 cm plate (50 mM HEPES pH 7.5, 150 mM NaCl, 10% glycerol, 1% Triton X-100, 1.5 mM MgCl_2_, 1 mM EDTA, 10 mM NaPPi, 100 mM NaF, + 10 µg/mL aprotinin, 10 µg/mL leupeptin and 1 mM AEBSF) and samples were centrifuged for 10 min at 4°C, 14000 x *g* to pellet cell debris. Lysates were precleared three times, each time using GST (100 μg) immobilized on glutathione Sepharose beads plus additional glutathione Sepharose beads (25 μl) for 1 hour at 4°C to remove non-specific binding proteins. Precleared supernants were analyzed by immunoblotting with anti-FLAG antibodies and were normalized for differences in FLAG-p85α protein expression by the addition of untransfected COS-1 cell lysates (prepared as above) to generate cell lysate samples containing similar levels of FLAG-p85α proteins. Lysates (5 μg in 200 μl) containing FLAG-p85α were used in each pull-down experiment with GST or GST-PTEN (5 μg each) immobilized on glutathione Sepharose beads. Samples were mixed for 30 minutes at 4°C and washed 5 times in 1 mL wash buffer (50 mM Tris, pH7.5, 150 mM NaCl, 1% NP-40). When using purified p85α proteins (250 μg in 1 mL 20 mM Tris pH 8, 100 mM NaCl), they were precleared by incubating with GST (200 μg) immobilized on glutathione Sepharose beads overnight at 4°C to remove non-specific binding proteins. Each pull-down experiment used GST or GST-PTEN (5 μg each) immobilized on glutathione Sepharose beads incubated with precleared p85α protein (5 μg in 1% milk in a total volume of 500 μl PBS (137 mM NaCl, 2.7 mM KCl, 4.3 mM sodium phosphate, 1.4 mM potassium phosphate, pH 7.3)) for 30 minutes at room temperature. Beads were washed five times in 1 mL wash buffer. In both cases, bound p85α was detected after resolving samples by SDS-PAGE and transferring protein to nitrocellulose, by immunoblotting with anti-FLAG antibody (1 μg/mL; Sigma #F3165) or anti- p85α antibody (1:200; EMD catalogue number 05 217) as indicated. Secondary antibodies linked to Infrared-dyes (IRDye800CW, IRDye680; LI-COR Biosciences) were used for detection and quantification using Odyssey software (v3.0). The results for all blots shown are representative of the results obtained from three independent experiments unless otherwise stated. Quantification of differences from wild type values were analyzed using a *t*-test with differences considered as statistically significant if *P* < 0.05.

### PTEN lipid phosphatase activity assay

A phosphate release assay was used to determine the effects of wild type and mutant p85α on PTEN activity as previously described [29]. Briefly, assays were performed by incubating His_6_-PTEN (1 µM) with Di-C8-PI3,4,5P_3_ lipid (200 µM; Echelon Biosciences) in the presence of wild type or mutant p85α (7.5 µM) in a final volume of 10 μl in phosphate release buffer (100 mM Tris-HCl pH 8.0, 1 mM DTT). All incubations were performed at 37°C for 20 min and reactions were stopped with the addition of 100 mM N-ethylmaleimide (15 μl; Sigma). For detection, 20 μl of the reaction was combined with 80 μl BIOMOL Green (Biorad) in a 96-well plate and the color was allowed to develop for 20 min at room temperature. The absorbance was measured at 620 nm with a microplate reader. Each data point was assayed in duplicate, and all experiments were repeated five times. Values for buffer controls were subtracted from those for experimental samples and reported relative to PTEN activity in the presence of wild type p85α. Assay results are expressed as means ± standard errors. One-way analysis of variance (ANOVA) with post hoc Bonferroni tests were used for multiple comparisons with differences considered as statistically significant if *P* < 0.05. As a further control, we added bovine serum albumin to PTEN in this assay and found no change to PTEN activity, verifying that the activation of PTEN activity is specific to p85 (data not shown).

### Circular dichroism

Circular dichroism spectra were recorded for the p85α BH domain mutants that showed reduced binding to PTEN as compared to the wild type p85α protein to ensure that protein folding was retained, as described previously [29].

### Protein-lipid overlay assay

Nitrocellulose-immobilized phospholipids (100 pmol per spot, PIP-Strips; Echelon Biosciences Inc.) were lysophosphatidic acid (LPA), lysophosphocholine (LPC), D-*myo*-phosphatidylinositol (PI), D-*myo*-phosphatidylinositol 3-phosphate (PI3P), D-*myo*-phosphatidylinositol 4- phosphate (PI4P), D-*myo*-phosphatidylinositol 5-phosphate (PI5P), L-α-phosphatidylethanolamine (PE), L-α-phosphatidylcholine (PC), sphingosine 1-phosphate (S1P), D-*myo*-phosphatidylinositol 3,4-bisphosphate (PI3,4,P_2_), D-*myo*-phosphatidylinositol 3,5-bisphosphate (PI3,5P_2_), D-*myo*-phosphatidylinositol 4,5-bisphosphate (PI4,5P_2_), D-*myo*-phosphatidylinositol 3,4,5-trisphosphate (PI3,4,5P_3_), L-α-phosphatidic acid (PA) and L- α -phosphatidylserine (PS). PIP strips were incubated in blocking buffer (3% [w/v] fatty acid free BSA (Sigma-Aldrich, Cat#A6003), in TBST (10 mM Tris pH 8.0, 150 mM NaCl, 0.01% Tween-20)) for 1 hour at room temperature with agitation. The PIP strips were then incubated overnight at 4°C with 37 nM purified p85α protein fragment in blocking buffer, unless otherwise indicated. PIP strips treated with no protein were used as a control. PIP strips were washed in TBST (6 times, 5 minutes each) and incubated for 1 hour at 20°C with a p85α BH domain-specific antibody (0.7 μg/ml in blocking buffer) [13]. They were washed as before and incubated for 90 minutes at 20°C with IRDYE 680 anti-rabbit secondary antibody (0.13 μg/ml in blocking buffer). The PIP strips were washed as described above. Proteins bound to the PIP strips were detected and visualized using a LI-COR Odyssey infrared scanner and analyzed with the Odyssey software (v3.0). Each result shown is representative of at least three independent experiments, each using a fresh lipid membrane.

### Rotational anisotropy

Reaction mixtures (70 µl), contained Bodipy-FL labelled phosphatidylinositol lipids (50 nm; Echelon Biosciences Cat#: C-00F6, C-03F6, C45F6, and C39F6) in 50 mM Tris pH 7.0, 150 mM NaCl, 1 mM EDTA buffer. To this mixture, p85α BH domain wild type or mutant proteins were added (16 – 1 μl additions; 0–78.6 μM); samples were mixed and incubated for 15 seconds at 25°C following each addition. Rotational anisotropy was measured using a QuantaMaster QM-4 fluorometer (Photon Technology International) with a single emission channel for 10 seconds. Samples were excited with vertically polarized light at 503 nm, and both vertical and horizontal emissions were monitored at 513 nm (2.5-nm band pass). Since none of the binding curves reached saturation, it was not possible to determine dissociation constants (*K*_d_) by fitting to a rectangular hyperbola, so we used Sigma Plot 11.2 software to highlight the slope of each dataset as a straight line.

### Protein purification, crystallization, structure determination and refinement

The purification, crystallization and structure determination for wild type and several mutants of the bovine p85α BH domain (105–319) has been described [29]. The p85α BH-R228E crystals were grown in 0.1 M Sodium Cacodylate pH 6.0, 1.5 M Li_2_SO_4_, and 4% (w/v) glycerol and the structure determined using X-ray diffraction data collected at the Canadian Light Source (CLS, Saskatoon, SK). Electron density was not present for residues 168–171 for both chains and residues 277–279 in Chain B. The atomic coordinates and diffraction intensities have now been deposited with the www Protein Data Bank and the corresponding deposition code is 6MRP.

### Protein modeling and docking

The crystallographic coordinates for the human PTEN (PDB ID# 1D5R) and the human p85α BH domain (PDB ID# 1PBW) were obtained from the Protein Data Bank (PDB). Figures were generated using the PyMOL Molecular Graphics System (Version 1.4.1 Schrödinger, LLC). We modeled the structure of inositol 1,3,4,5-tetrakisphosphate (PDB ID# 1BWN) [45] in the newly identified potential binding pocket in the bovine p85α BH domain in *Coot*, aided by the position of a vanadate ion and a tartrate ion observed in the active site of PTEN where the active site cysteine nucleophile (Cys124) forms a disulfide with Cys71 [30]. The 3-phosphate of inositol 1,3,4,5-tetrakisphosphate was approximately overlaid on the position of the bound vanadate and 4-phosphate was positioned near the tartrate C4 carboxylate.

Docking analysis was performed using the ClusPro 2.0 server [31–34]. The previously solved PTEN crystal structure (PDB ID# 1D5R) was entered as the receptor molecule, while the A Chain from our solved bovine p85α (105–319) crystal structure [29] or the human p85α BH domain (PDB ID# 1PBW) were used as the ligand structures. Residues set to be attraction residues were based on our experimental evidence and were p85α residues D168, E212, E218, K224, K225, R228, K249, S231, H234, L238, and Q241, and PTEN residues K342 and K344. Resulting docking models were analyzed in PyMOL to determine the most physiologically relevant models determined. Parameters for determining physiological relevance included proximity of p85α to the PTEN catalytic pocket, involvement of experimentally determined residues in the interaction interface, and orientation of proteins to allow for inclusion of other domains, and expected orientations with respect to lipid membranes. To locate the position of Rab5 relative to the PTEN – p85α BH domain docked structure, we overlaid the individual structures of Rab5 and the bovine p85α BH domain with the known crystal structure of the Cdc42GAP – Cdc42 complex as described previously [29].

## SUPPLEMENTARY MATERIALS FIGURES AND TABLE



## Abbreviations

GST: glutathione S-transferase
PASE: phosphatase domain
PCR: polymerase chain reaction
PDK1: 3-phosphoinositide-dependent protein kinase 1
PDZB: post synaptic density protein binding region, *Drosophila* disc large tumor suppressor, and zonula occludens-1 protein PI3,4,5P_3_, phosphatidylinositol 3,4,5-trisphosphate
PI3K: phosphatidylinositol 3-kinase
PI4,5P_2_: phosphatidylinositol 4,5-bisphosphate
PMB: plasma membrane binding region
REG: regulatory domain
