# Supplementary material for: Insight into the PTEN – p85α interaction and lipid binding properties of the p85α BH domain
Source: Oncotarget. 2018 Dec 11;9(97):36975–92. doi: 10.18632/oncotarget.26432 (PMC6319338; doi:10.18632/oncotarget.26432)
Supplement: Supplementary file 1 [file oncotarget-09-36975-s001.pdf]

Insight into the PTEN – p85α interaction and lipid binding properties of the p85α BH domain

SUPPLEMENTARY MATERIALS

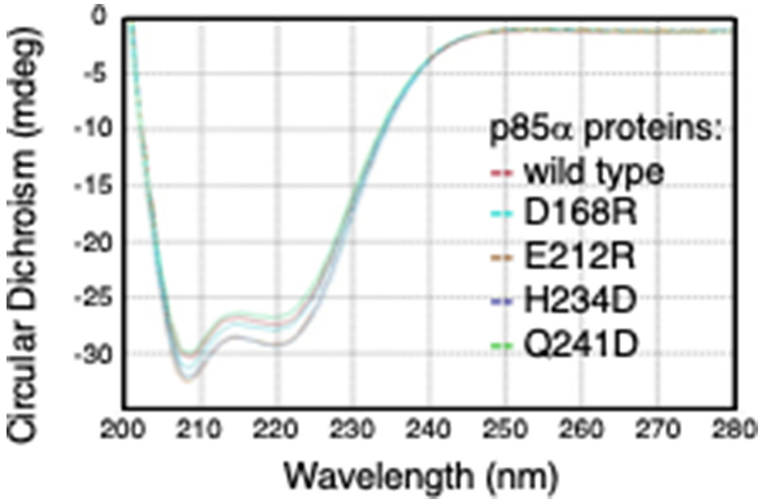

Supplementary Figure 1: Characterization of key p85α mutants folding structure. Circular dichroism spectra comparing purified mutant p85α proteins to the control p85α wild type protein to ensure secondary and tertiary structure is retained.

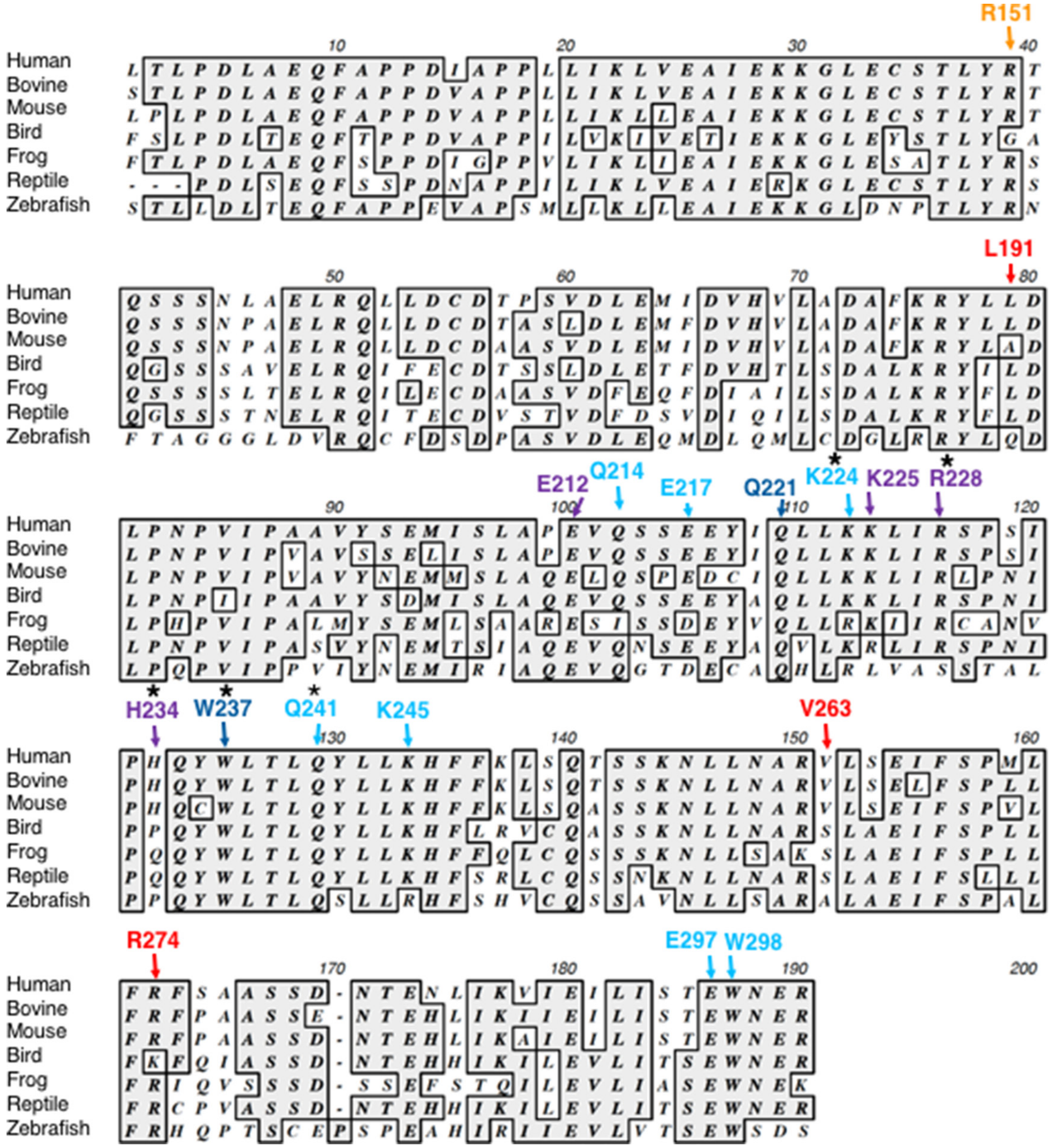

Supplementary Figure 2: Sequence alignment of p85α BH domains from different vertebrates. The p85α BH domains of Human (accession number P27986; residues 113–301), Bovine (accession number P23727; residues 113–301), Mouse (accession number P26450; residues 113–301), Bird (Zebra Finch: Taeniopygia Guttata; accession number H0YXT8; residues 113–301), Frog (African clawed frog: Xenopus Laevis; accession number Q8UUU2; residues 112–300), Reptile (Carolina anole: Anolis Carolinensis; accession number Q9H0H5; residues 349–539) and Zebrafish (Danio Rerio; accession number Q9H0H5; residues 349–539) were aligned. The location of p85α BH mutations that decreased PTEN binding experimentally and that both docking models position to form direct contacts with PTEN are shown in purple. The docking model also identified several new p85α BH domain residues that are predicted to form direct contacts with PTEN, but were not tested for their impacts on PTEN binding (sky-blue if predicted to be important from the both human and bovine p85α BH docking models with PTEN; cyan if only predicted to be important in one of the docking models). Red and orange residues mark the Rab5 binding site. The five residues involved in binding the two sulfate molecules are marked with an asterisk (*).

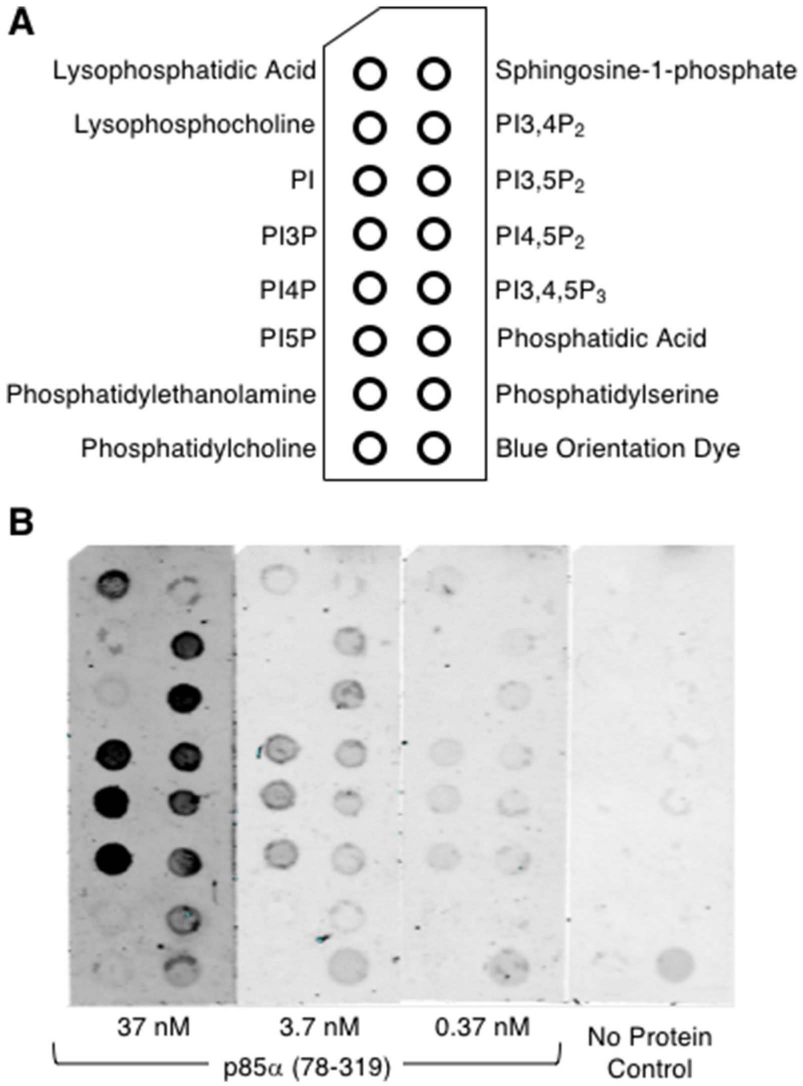

Supplementary Figure 3: Concentration-dependent binding of the p85α (78–319) protein to phospholipids. (**A**) PIP strip schematic. (**B**) PIP strips containing various lipids bound to nitrocellulose were probed with decreasing concentrations of p85α (78–319): 37, 3.7, 0.37 nM. PIP strips probed with no protein were used for control. Bound protein was detected using an anti-p85α-BH primary antibody, with an infrared secondary antibody and visualized using a LICOR Odyssey infrared scanner.

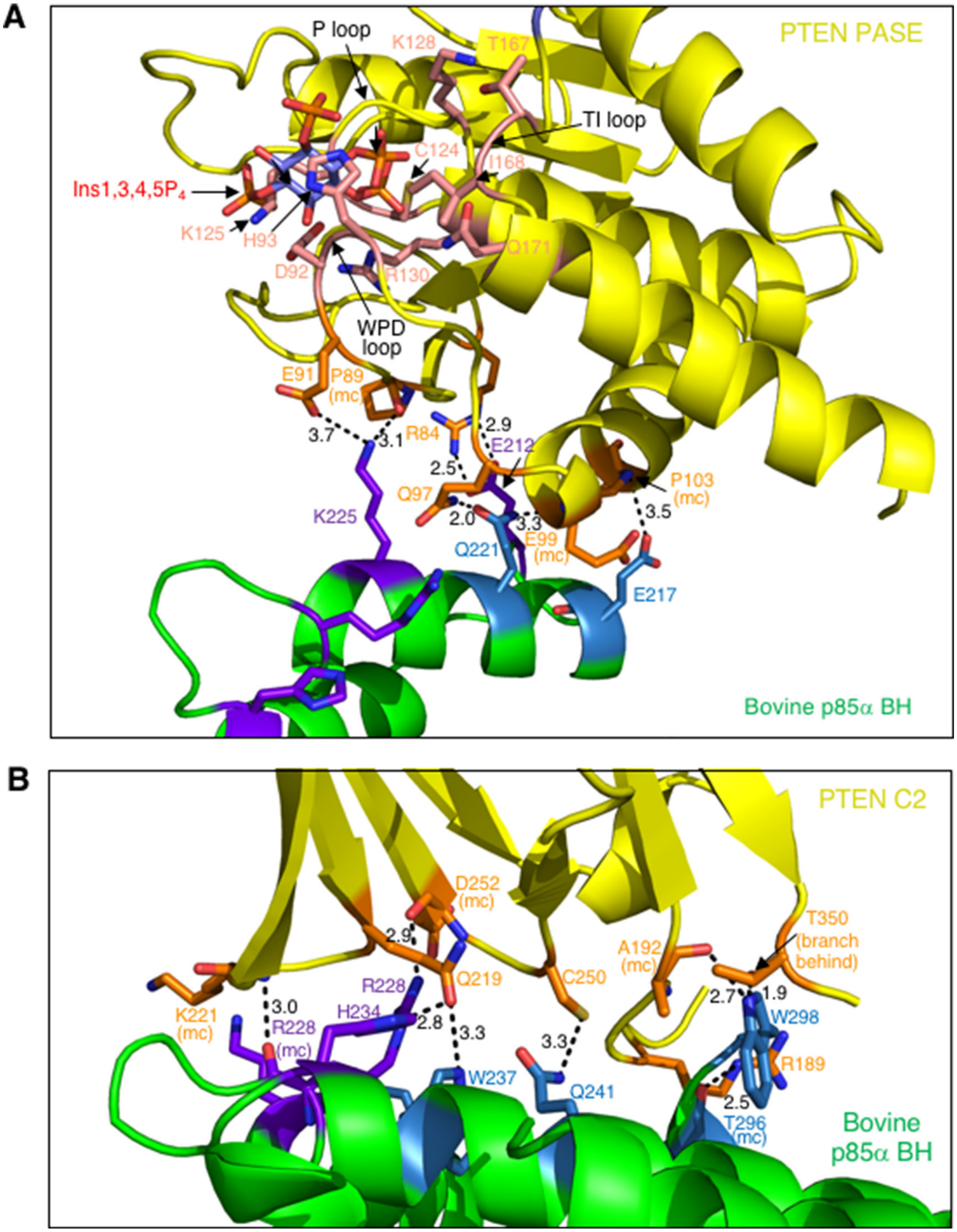

Supplementary Figure 4: Close-up view of docking model for bovine p85α BH domain monomer and PTEN, expanded from Figure 7A. (**A–B**) Close-up of the direct contacts between the bovine p85α BH domain (green; PDB ID# 6D81) and the PTEN (yellow; PDB ID# 1D5R) C2 domain (**A**) or PASE domain (**B**; p85α residues F122 – V127 are hidden). PTEN active site residues (salmon) are modeled with bound inositol 1,3,4,5-tetrakisphosphate ($Ins1,3,4,5P_4$; multi-colored). PTEN residues involved in making direct contacts with the p85α BH domain are shown in orange. The bovine p85α BH domain residues involved in direct contacts with PTEN that were also experimentally tested and determined to be important for PTEN binding are shown in purple, with additional contact residues identified by the docking model shown in sky blue. Bond distances are shown in Å. (mc) = main chain.

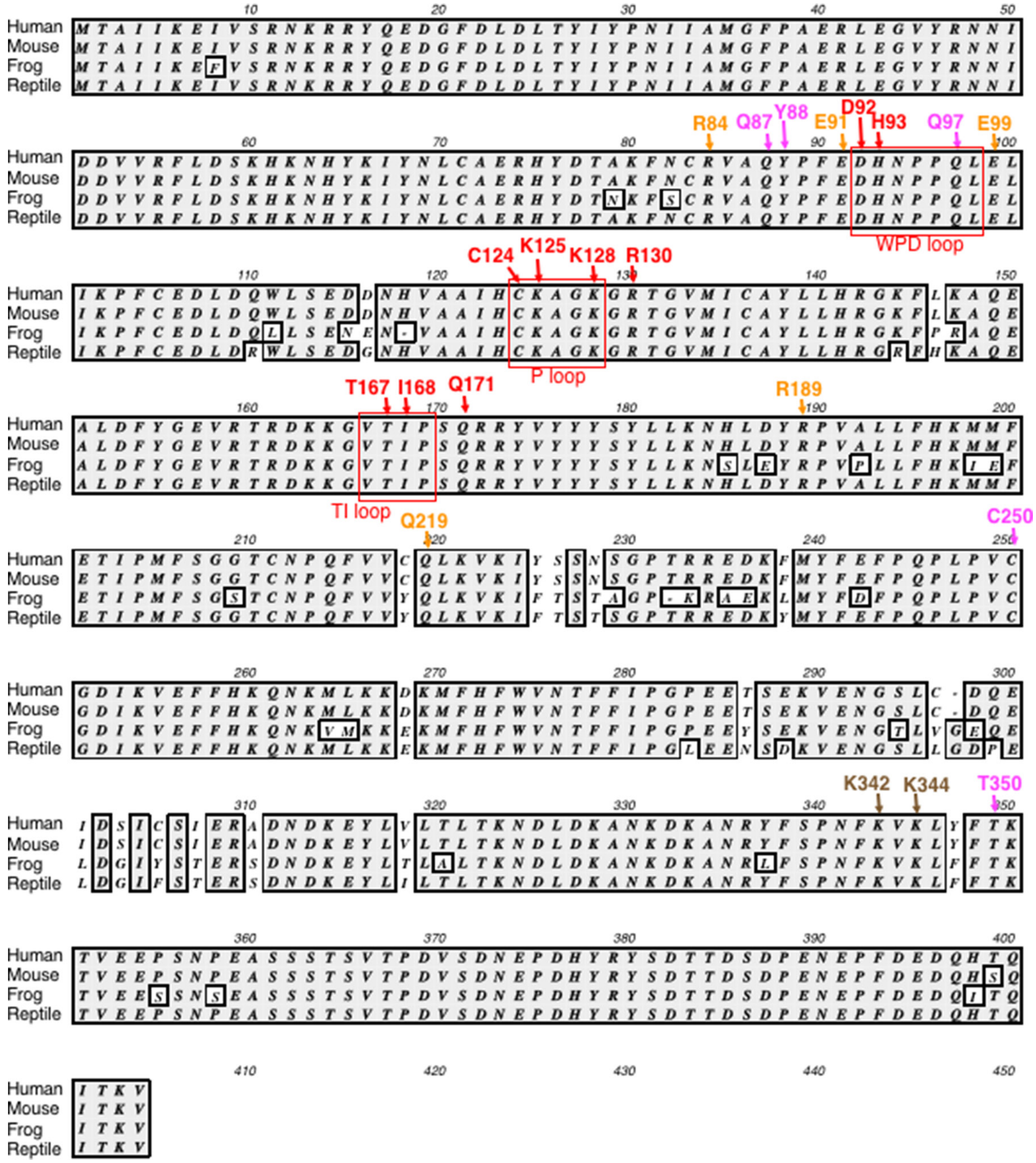

Supplementary Figure 5: Sequence alignment of PTEN from different vertebrates. PTEN sequences from Human (accession number P60484), Mouse (accession number O08586), Frog (African clawed frog: Xenopus Laevis; accession number Q9PUT6) and Reptile (Carolina anole: Anolis Carolinensis; accession number H9GLY0) were aligned. The location of PTEN residues involved in direct side-chain binding contacts with the p85α BH domain within both docking models are shown in orange. PTEN residues predicted to be important in only one of the docking models are shown in pink. Red boxes indicate catalytic loops with key catalytic PTEN residues shown in red. The PTEN residues that showed decreased binding to full-length p85α when mutated are indicated in brown.

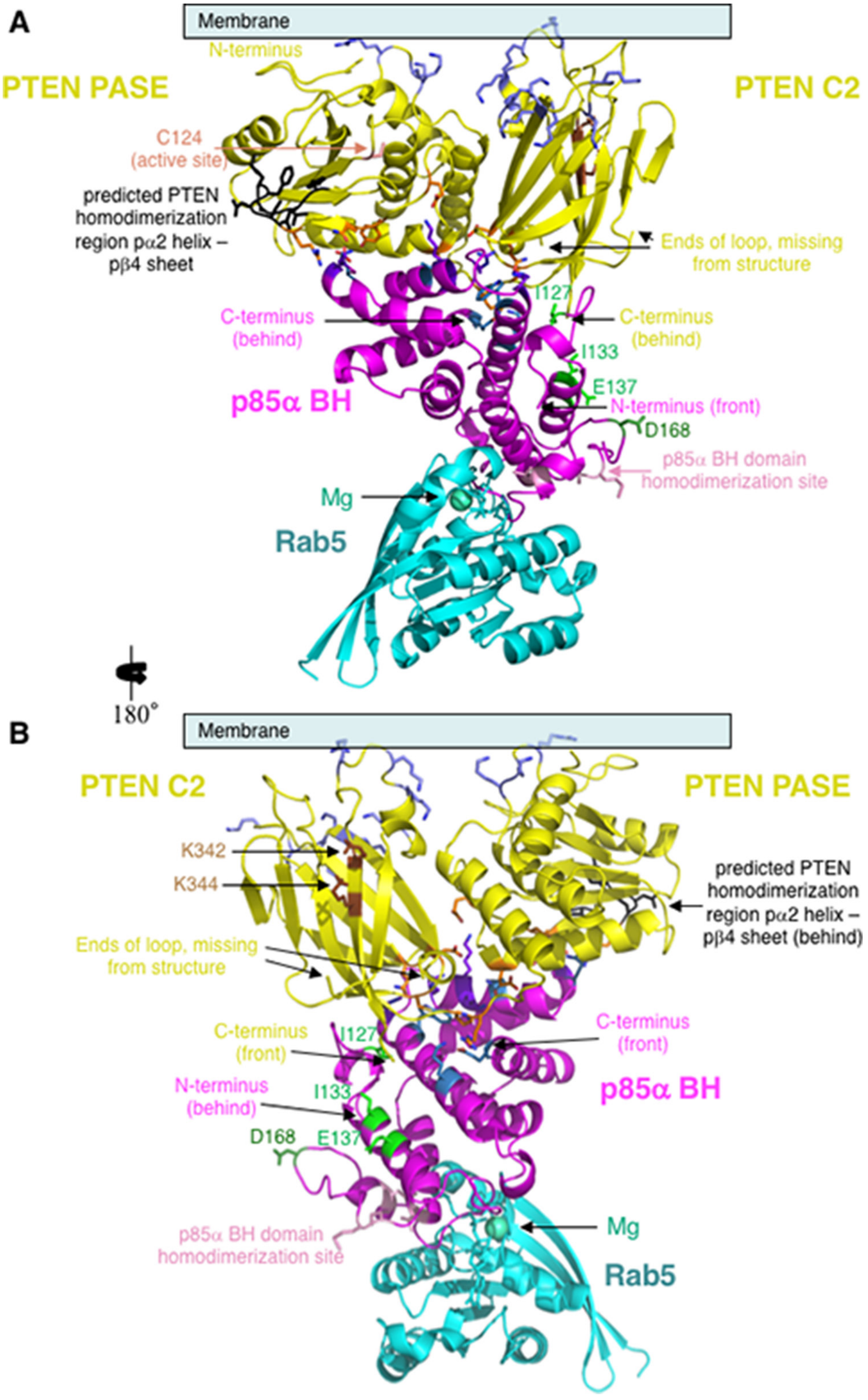

Supplementary Figure 6: PTEN (yellow; residues 7–353, Δ286–309) – p85α BH domain (human; magenta; residues 115–298) docked model showing the polypeptide ends and the relative position of the bound GTPase Rab5 (cyan). (**A–B**) Two views of the complex: the PTEN basic residues important for membrane binding are shown in slate blue (R161, K163, K164; K260, K263, K266, K267, K269; K327, K330, K332, R335). The region of PTEN predicted to mediate PTEN – PTEN homodimerization is shown in black (pα2 helix – pβ4 sheet). The p85α BH domain residues involved in p85α BH – BH homodimerization are shown in pink (L161, M176, I177, V181). The locations of three p85α BH domain residues previously implicated in PTEN binding (light green; I127, I133, E137) and a nearby residue important for PTEN binding (dark green; D168; Figure 3B) are also shown.

Supplementary Table 1: Comparison of the predicted direct binding interactions between the docking models for the human p85α BH domain and PTEN, and the bovine p85α BH domain and PTEN, as determined using ClusPro docking software to model these complexes

Human p85α	*PTEN*	*Bovine p85α*	*PTEN*	*PTEN domain*
E212 (sc O)	Q87 (sc N)	E212 (sc O)	R84 (sc N)	PASE
Q214 (sc O)	R84 (sc N)			PASE
Q214 (sc N)	Y88 (sc O)			PASE
		E217 (O)	P103 (mc N)	PASE
Q221 (sc N)	E99 (sc O)	Q221 (sc N)	E99 (mc O)	PASE
		Q221 (sc O)	Q97 (sc N)	PASE
K224 (sc N)	E99 (sc O)			PASE
K225 (sc N)	E91 (sc O)	K225 (sc N)	E91 (sc O)	PASE
		K225 (sc N)	P89 (mc O)	PASE
R228 (sc N)	D252 (mc O)	R228 (sc N)	D252 (mc O)	C2
R228 (sc N)	C250 (mc O)	R228 (mc O)	K221 (mc N)	C2
H234 (sc N)	Q219 (sc O)	H234 (sc N)	Q219 (sc O)	C2
W237 (sc N)	Q219 (sc O)	W237 (sc N)	Q219 (sc O)	C2
		Q241 (sc N)	C250 (sc S)	C2
K245 (sc N)	P190 (mc O)			C2
E297 (sc O)	R189 (sc N)	T296 (mc O)	R189 (sc N)	C2
		W298 (sc N)	T350 (sc O)	C2
		W298 (sc N)	A192 (mc O)	C2

sc = side chain
mc = main chain
O = oxygen
N = nitrogen
